# Effect of Community-Initiated Kangaroo Mother Care on Postpartum Depressive Symptoms and Stress Among Mothers of Low-Birth-Weight Infants: A Randomized Clinical Trial

**DOI:** 10.1001/jamanetworkopen.2021.6040

**Published:** 2021-04-22

**Authors:** Bireshwar Sinha, Halvor Sommerfelt, Per Ashorn, Sarmila Mazumder, Sunita Taneja, Deepak More, Rajiv Bahl, Nita Bhandari

**Affiliations:** 1 Centre for Health Research and Development, Society for Applied Studies, New Delhi, India; 2 Center for Child Health Research, Faculty of Medicine and Health Technology, Tampere University Hospital, Tampere University, Tampere, Finland; 3 DBT/Wellcome India Alliance, Hyderabad, India; 4 Centre for Intervention Science in Maternal and Child Health, Department of Global Public Health and Primary Care, University of Bergen, Bergen, Norway; 5 Cluster for Global Health, Division for Health Services, Norwegian Institute of Public Health, Oslo, Norway; 6 Clinical and Research Laboratories, Society for Applied Studies, New Delhi, India; 7 Department of Maternal, Newborn, Child and Adolescent Health, World Health Organization, Geneva, Switzerland

## Abstract

**Importance:**

Approximately 1 in 5 women in low- and middle-income countries experience postpartum depression, and the risk is higher among mothers of low-birth-weight (LBW) infants. Kangaroo mother care (KMC) is effective in improving survival among LBW infants, but the benefits of KMC for mothers are not well described.

**Objective:**

To estimate the effects of community-initiated KMC (ciKMC) on maternal risk of moderate-to-severe postpartum depressive symptoms and on salivary cortisol concentration, a biomarker of stress.

**Design, Setting, and Participants:**

This was an unmasked, parallel-group, individually randomized clinical trial. Participants included 1950 mothers of stable LBW infants (weighing 1500-2250 g) in rural and semiurban low-income populations in North India enrolled between April 2017 and March 2018. Data analysis was performed from January to July 2020.

**Interventions:**

Eligible participants were randomly assigned to the intervention or control group by block randomization. The mothers in the intervention group were supported to practice ciKMC until 28 days after birth or until the infant wriggled out of the KMC position (ie, was no longer staying in the KMC position). The intervention included promotion and support of skin-to-skin contact and exclusive breastfeeding through home visits.

**Main Outcomes and Measures:**

Postpartum depressive symptoms at the end of the neonatal period were measured using the Patient Health Questionnaire–9, with a score of 10 or higher used to identify moderate-to-severe depressive symptoms. Salivary cortisol concentration was measured in a subsample of 550 mothers before and after breastfeeding on day 28 after birth.

**Results:**

Of the 1950 participants (mean [SD] age, 23 [3.5] years), outcome assessment was completed for 974 of 1047 participants (93%) in the intervention group and 852 of 903 participants (94%) in the control group. Sixty-four percent of participants (1175 of 1826 participants) belonged to the lowest 3 wealth quintiles. The proportion of mothers with moderate-to-severe postpartum depressive symptoms was 10.8% (95% CI, 8.9%-12.9%; 105 of 974 mothers) in the intervention group vs 13.6% (95% CI, 11.4%-16.1%; 116 of 852 mothers) in the control group. The adjusted relative risk of moderate-to-severe maternal postpartum depressive symptoms was 0.75 (95% CI, 0.59-0.96), or an efficacy of 25%. There was no difference in day-28 salivary cortisol concentration between the ciKMC and control group mothers before or after breastfeeding. The analysis estimated that supporting 36 mothers to perform KMC at home would prevent 1 mother from experiencing moderate-to-severe postpartum depressive symptoms.

**Conclusions and Relevance:**

These findings suggest that ciKMC practice may substantially reduce the risk of moderate-to-severe maternal postpartum depressive symptoms. This evidence supports KMC as an intervention to be incorporated in essential newborn care programs in low- and middle-income settings.

**Trial Registration:**

Clinical Trials Registry–India Identifier: CTRI/2017/04/008430

## Key Points

**Question:**

Does the practice of community-initiated kangaroo mother care (ciKMC), an intervention encompassing skin-to-skin-contact and exclusive breastfeeding, during the neonatal period reduce the risk of moderate-to-severe postpartum depressive symptoms among mothers of low-birth-weight (LBW) infants?

**Findings:**

In a randomized clinical trial that included 1950 mothers of stable LBW infants from low-income areas in India, the practice of ciKMC resulted in a 25% reduction in the relative risk of moderate-to-severe depression at 4 weeks after delivery. The analysis estimated that supporting 36 mothers to perform KMC at home would prevent 1 mother from experiencing moderate-to-severe postpartum depressive symptoms.

**Meaning:**

These findings suggest that ciKMC can have substantial benefits for maternal mental health, beyond improving the survival of LBW infants.

## Teaser

This randomized clinical trial estimates the effect of community-initiated kangaroo mother care on maternal risk of moderate-to-severe postpartum depressive symptoms among mothers of low-birth-weight (LBW) infants.

## Introduction

Postpartum depression^1,2^ affects quality of life and long-term psychological health in mothers and can also adversely affect mother-child interaction, breastfeeding, infant growth, and development.^3,4,5,6,7^ Pooled estimates from 53 studies in 23 low- and middle-income countries reported the prevalence of postpartum depression to be 19% (95% CI, 16%-23%).^8^ A meta-analysis^9^ that included 38 studies in India estimated the prevalence of postpartum depression to be 22% (95% CI, 19%-25%). The prevalence of depression is reported to be higher among mothers of preterm compared with full-term infants in the first 12 weeks after birth.^10,11^

Several psychosocial and psychological interventions have been evaluated for their effect on the risk of postpartum depression. These include postpartum home visits by professionals, telephone-based peer support, and interpersonal psychotherapy. Although promising, they are not widely implemented in the Indian public health system.^12^ Kangaroo mother care (KMC), an intervention encompassing skin-to-skin contact (SSC) and exclusive breastfeeding, reduces the risk of death and severe infection in low-birth-weight (LBW) infants and is recommended by the World Health Organization and the Government of India.^13,14,15^ In a large randomized clinical trial^16^ in India among 8402 LBW infants, promotion of community-initiated KMC (ciKMC) improved postenrollment neonatal survival by 30% and infant 6-month survival by 25%. KMC could reduce the risk of postpartum depressive symptoms through mother-infant bonding and possibly via the release of maternal oxytocin and lowering of cortisol secretion.^17,18,19^ Data from observational and quasi-experimental studies^20,21,22,23^ suggest such a beneficial effect on mothers, but conclusive evidence is lacking.

Our primary objective was to test the hypothesis that the practice of ciKMC during the neonatal period can reduce the risk of moderate-to-severe maternal postpartum depressive symptoms. As a secondary objective, we estimated the effect of ciKMC on maternal salivary cortisol concentration, a biomarker of stress, at the end of neonatal period.

## Methods

### Study Design and Participants

This unmasked, parallel-group, individually randomized clinical trial was developed as a substudy within a larger primary trial where the effect of ciKMC on neonatal and early infant mortality was estimated (ClinicalTrials.gov identifier: NCT02653534).^16,24^ Here, we report the outcomes related to maternal mental health (ie, postpartum depressive symptoms) and stress as measured by cortisol. The study was conducted in rural and semiurban low-income populations of Faridabad and Palwal districts in Haryana, India.

Ethics approval was obtained from the institutional ethics committee at the Centre for Health Research and Development, Society for Applied Studies, New Delhi, India, and the Regional Committee for Medical and Health Research Ethics in Norway. For eligible mothers, written informed consent was obtained in the local language prior to enrollment (see the Trial Protocol in Supplement 1). The study is reported as per the Consolidated Standards of Reporting Trials (CONSORT) reporting guideline.^25^

**Figure 1.**
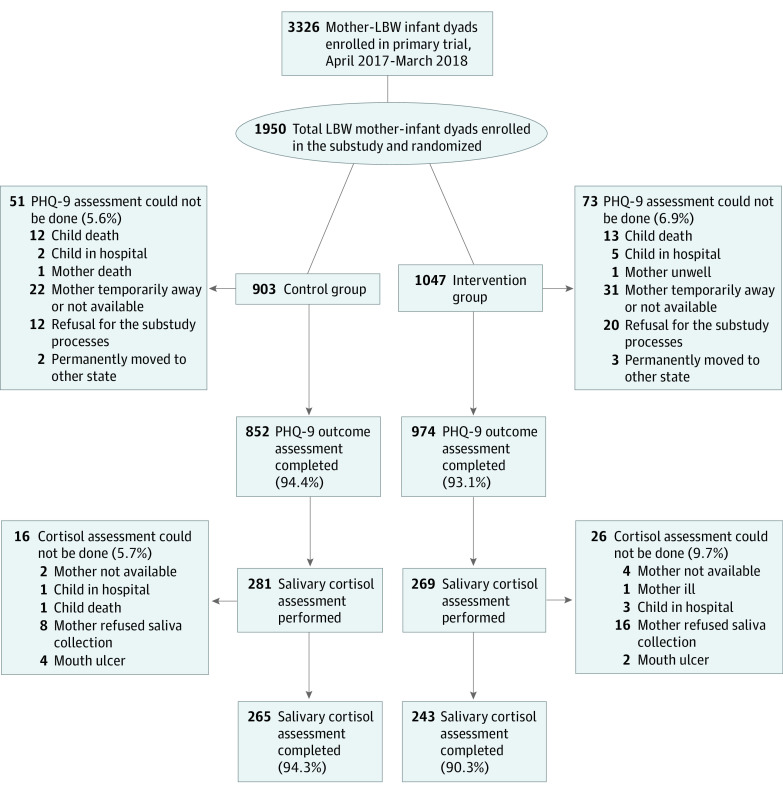
Flowchart of Participants LBW indicates low birth weight; PHQ-9, Patient Health Questionnaire–9.

As part of the primary trial, pregnant women were followed up by a surveillance team until delivery. Newborn infants weighing 1500 to 2250 g and their mothers screened within 72 hours of birth were eligible to be included in the trial, unless KMC had already been initiated in a birth facility or infants were unable to feed, had breathing problems, had gross congenital malformations, or had less than normal movements.^26^ Mothers not living with their infants and those intending to move away over the next 6 months were excluded. In our substudy, we excluded mothers of twins or triplets. For feasibility reasons, we made an a priori decision to restrict enrollments to a maximum of 6 per day, randomly selected from those enrolled in the primary trial. Between April 2017 and March 2018, 1950 of 3326 mothers enrolled in the primary trial were included in this substudy for evaluation of postpartum depressive symptoms (Figure 1).

### Randomization and Masking

The randomization list was prepared by an independent statistician using random permuted blocks of variable size. Allocation of participant identification number was done by an off-site randomization coordinator using serially numbered opaque sealed envelopes. To minimize contamination between trial groups, if a previously enrolled infant was allocated to the intervention group, the next eligible infant belonging to the same household was also allocated to intervention. If a previously enrolled infant was allocated to the control group, the next eligible infant in the same household was assigned to intervention or control as per the randomization sequence. Further details of methods of the primary trial have been published elsewhere.^16,24^

### Intervention and Usual Care

The ciKMC intervention comprised promotion and support of SSC and exclusive breastfeeding. Infant-mother dyads allocated to the ciKMC group were visited at home by an intervention delivery team consisting of a pair of trained workers to initiate and support KMC. The team observed breastfeeding and promoted SSC by using photographs and the local term *chaati se chipkaana*, which means *sticking the baby to the chest*. Mothers were counseled to practice SSC as long as possible during day and night with the assistance of other family members and were taught to daily record the duration of SSC. The team visited the families on days 1, 2, 3, 5, 7, 10, 14, 21, and 28 after birth to observe and address any problems related to SSC and/or breastfeeding; the duration of the visits ranged from 30 to 45 minutes. Visits continued until the infant wriggled out from the KMC position and no longer accepted SSC or until 28 days of age, whichever was earlier.^24,26^ Referral of ill infants in both trial groups was facilitated through government-accredited social health activists. All infants in the intervention and control groups received usual care (ie, home-based postnatal care visits by accredited social health activists as implemented through the health system).^27^

### Outcomes

Our primary outcome was moderate-to-severe postpartum depressive symptoms at the end of the neonatal period (day 28 after birth) measured using a pretested Hindi version of the validated Patient Health Questionnaire–9 (PHQ-9).^28,29^ Secondary outcomes were any depressive symptoms, PHQ-9 score, and maternal salivary cortisol concentration at the end of neonatal period. Postpartum depressive symptoms were defined as PHQ-9 scores of 10 or higher for moderate-to-severe symptoms, 5 to 9 for mild symptoms, and 0 to 4 for no or minimal symptoms.^28^ A PHQ-9 score of 5 or higher defined any depressive symptoms. The sensitivity and specificity of a PHQ-9 score 10 or higher to diagnose major depression in the postpartum period are reported to be approximately 80% and greater than 90%, respectively.^29,30^

### Assessments

Study workers were trained in good clinical practice.^31^ Reported duration of SSC and exclusive breastfeeding were captured by an independent outcome ascertainment team at the end of the neonatal period for all study participants. For the PHQ-9 interviews, an independent team of health workers was trained and assessed by a clinical psychologist. The PHQ-9 assessment was done after day 28, but not later than day 42 after birth. Supervisors conducted random quality checks in 1% of the assessments. Women with PHQ-9 scores 10 or higher were referred to health facilities.

We collected salivary specimens from the first 550 mothers on day 28 after birth to measure salivary cortisol. To account for diurnal fluctuations and variability related to breastfeeding, the specimens were collected before and after breastfeeding, not later than noon. The cortisol concentration was estimated by a Cortisol Enzyme Immunoassay Kit (Salimetrics) using an automated system (Twin Plus, Evolis) at Clinical and Research Laboratories, Society for Applied Studies, New Delhi. The kit performed within acceptable limits for accuracy, precision, and linearity for values between 1.0 μg/dL and 0.038 μg/dL (to convert cortisol to nanomoles per liter, multiply by 27.588). Specimens with values less than or equal to 0.037 μg/dL were assigned the lowest acceptable value.

### Statistical Analysis

Assuming 90% power, 95% confidence, and 10% attrition and to be able to identify a minimum of 30% relative reduction from a 19% proportion of mothers with moderate-to-severe postpartum depressive symptoms in the control group,^9^ the total sample size required was 1942 participants according to a 2-sided test (see the Statistical Analysis Plan in Supplement 1). Analyses were conducted on an intent-to-treat basis using Stata statistical software version 16 (StataCorp). We estimated gestational age from ultrasonography reports, hospital records, or maternal recall, whichever were available, in the given order of preference.

We estimated relative risks (RRs) with 95% CIs for maternal moderate-to-severe or any depressive symptoms between the study groups using generalized linear models of the binomial family with a log-link. The efficacy of the intervention against maternal moderate-to-severe depressive symptoms was calculated as (1 − RR) × 100. We conducted multinomial logistic regression to estimate the effect of ciKMC on the risk of different categories of maternal depressive symptoms with no or minimal depressive symptoms (PHQ-9 scores of 0-4) as the reference. To compare salivary cortisol levels, we used the Wilcoxon rank-sum test.

Following CONSORT 2010 guidelines,^32^ our approach was to present both unadjusted and adjusted results while avoiding overadjustment. Wealth quintile, mother age, mother education, and birth order were the potentially confounding covariates associated with the primary outcome at *P* < .10 in univariable analysis. To avoid overadjustment,^33^ we included the potential confounding factors in the final multivariable regression model only if they were unequally distributed between the study groups at baseline, a priori defined as a relative difference of greater than 10%.^34^ We used the likelihood ratio test based on deviance statistics to assess goodness-of-fit of the model^35^ Design effects of infant-mother dyads enrolled from a single household were accounted for using Stata’s robust variance estimator (vce) option. To quantify any biological interaction between ciKMC and preterm birth, we estimated the relative excess risk due to interaction^36^ using the icp command.

We conducted exploratory subgroup analyses to estimate whether the effect of ciKMC on postpartum depressive symptoms was different in mothers with preterm birth (<37 weeks gestation) compared with full-term birth (≥37 weeks gestation). Data analysis was performed from January to July 2020.

## Results

Of the 1950 enrolled participants (mean [SD] age, 23 [3.5] years), we completed PHQ-9 assessments for 93% of mothers (974 of 1047 participants) in the intervention group and 94% of mothers (852 of 903 participants) in the control group (Figure 1). In both study groups, 64% of participants (1175 of 1826 participants) belonged to the lower 3 wealth quintiles. Other baseline characteristics were well balanced across the study groups (Table 1) except for high birth order (ie, birth order ≥5), where the relative difference between study groups was 26%.

**Table 1.** Baseline Characteristics of Participants in the Control and Intervention Groups

Variables	Participants, No. (%)	
	Control (n = 852)	ciKMC (n = 974)
Household characteristics		
Wealth quintiles, poor		
Least	146 (17.1)	167 (17.2)
Less	157 (18.4)	181 (18.6)
Poor	178 (20.9)	204 (20.9)
Very	204 (23.9)	229 (23.5)
Most	167 (19.6)	193 (19.8)
Family social class		
General	191 (22.4)	200 (20.5)
Other class^a^	389 (45.7)	434 (44.6)
Scheduled caste or tribe	272 (31.9)	340 (34.9)
Type of family		
Nuclear	250 (29.3)	312 (32.0)
Joint	602 (70.7)	661 (68.0)
Family members, mean (SD), No.	7.4 (3.2)	7.7 (3.5)
Maternal and paternal characteristics^b^		
Maternal age, mean (SD), y	23.1 (3.5)	23.4 (3.6)
Maternal education		
None	283 (33.2)	341 (35.0)
Duration of education, mean (SD), y	6.3 (5.2)	6.1 (5.2)
Mother working outside home	11 (1.3)	14 (1.4)
Father’s age, mean (SD), y	26.5 (4.6)	26.7 (5.2)
Father’s education		
None	128 (15.0)	150 (15.4)
Duration of education, mean (SD), y	8.5 (4.8)	8.3 (4.8)
Father not working	51 (6.0)	50 (5.1)
Birth-related characteristics		
Place of delivery		
Home	129 (15.1)	150 (15.4)
Government facility	511 (60.0)	569 (58.4)
Private facility	212 (24.9)	255 (26.2)
Cesarean delivery	18 (2.1)	18 (1.9)
Birth order^b^		
1	343 (40.3)	367 (37.7)
2-4	450 (52.8)	515 (52.9)
≥5	59 (6.9)	91 (9.4)
Infant characteristics		
Female	470 (55.2)	557 (57.2)
Weight at enrollment, median (IQR), kg	2.1 (2.0-2.2)	2.1 (2.00-2.2)
Weight at enrollment, kg		
1.50-1.79	44 (5.2)	52 (5.3)
1.80-1.99	160 (18.8)	182 (18.7)
2.00-2.25	648 (76.1)	740 (76.0)
Gestational age, mean (SD), wk^c^	35.8 (2.0)	35.8 (2.0)
Preterm <37 wk^c^	535 (62.8)	619 (62.8)

Abbreviations: ciKMC, community-initiated kangaroo mother care; IQR, interquartile range.

^a^ Refers to any class not included in the general group or a scheduled caste or tribe.

^b^ Baseline information on parental characteristics and birth order is not available for 1 participant in the intervention group.

^c^ A total of 1253 of 1826 mothers (68.6%) underwent an ultrasonography.

In the intervention group, 99% of the mothers (971 of 974 mothers) reported practicing SSC during the neonatal period vs 4% (33 of 852 mothers) in the control group. In the intervention group, the median (interquartile range) age of the infant at ciKMC initiation was 48 (23-72) hours; the mean (SD) duration of practicing SSC was 27.5 (3.9) days with a mean (SD) of 12.0 (3.7) hours per day. Approximately 2% of mothers continued SSC beyond the 28-day period. Exclusive breastfeeding prevalence (24-hour recall) at day 28 was 88% in the intervention group (859 of 974 participants) vs 57% (486 of 852 participants) in the control group. The number of home visits by accredited social health activists for postnatal care were similar in the 2 trial groups (eTable 1 in Supplement 2).

**Figure 2.**
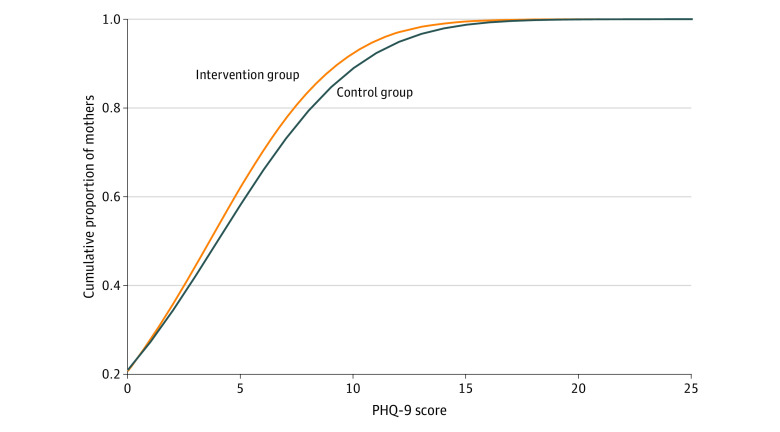
Cumulative Frequency Plot Showing Patient Health Questionnaire–9 (PHQ-9) Scores of Mothers in Control and Intervention Groups

The proportion of mothers with moderate-to-severe postpartum depressive symptoms was 10.8% (95% CI, 8.9%-12.9%; 105 of 974 mothers) in the intervention group vs 13.6% (95% CI, 11.4%-16.1%; 116 of 852 mothers) in the control group (Table 2). The RR for moderate-to-severe postpartum depressive symptoms adjusted for birth order categories and taking household clustering into account was 0.75 (95% CI, 0.59-0.96), corresponding to an efficacy of 25% (95% CI, 4%-41%); the unadjusted RR was 0.79 (95% CI, 0.62-1.01). Additional adjustments for wealth quintile, mother age, and mother education changed the adjusted effect estimate only negligibly (data not shown). Multinomial regression showed a similar adjusted effect size of the intervention on the risk of moderate-to-severe depressive symptoms (RR, 0.73; 95% CI, 0.54-0.98), but no effect on mild depressive symptoms (RR, 1.00; 95% CI, 0.79-1.26) (eTable 2 in Supplement 2). The absolute risk difference for moderate-to-severe postpartum depressive symptoms was 2.8% (95% CI, 0.1%-5.8%), corresponding to a number needed to treat of 36 mother-infant dyads (95% CI, 17-1000 dyads). The median (interquartile range) PHQ-9 scores were 2 (0-5) in the intervention group and 2 (0-6) in the control group. The cumulative frequency plot showed a left-shift of the PHQ-9 scores among ciKMC group compared with the control group mothers (Figure 2), suggesting that at any given PHQ-9 score cutoff, a lower proportion of mothers in the ciKMC group were above the cutoffs (eTable 3 in Supplement 2).

**Table 2.** Effect of ciKMC on Maternal Postpartum Depressive Symptoms Among Study Participants

Outcome variable	Participants, No./Total No. (%)		RR (95% CI)	
	Control	ciKMC	Unadjusted	Adjusted^a^
Moderate-to-severe depressive symptoms^b^				
All mothers	116/852 (13.6)	105/974 (10.8)	0.79 (0.62-1.01)	0.75 (0.59-0.96)^c^
Subgroup				
Mothers of preterm infants	80/535 (15.0)	68/612 (11.1)	0.73 (0.55-1.00)	0.71 (0.52-0.96)
Mothers of full-term infants	36/317 (11.4)	37/362 (10.2)	0.90 (0.58-1.39)	0.86 (0.56-1.34)
Any depressive symptoms^d^				
All mothers	275/852 (32.3)	294/974 (30.2)	0.94 (0.82-1.07)	0.92 (0.81-1.05)
Subgroup				
Mothers of preterm infants	183/535 (34.2)	195/612 (31.2)	0.74 (0.55-1.00)	0.92 (0.78-1.09)
Mothers of full-term infants	92/317 (29.0)	99/362 (27.3)	0.90 (0.58-1.38)	0.91 (0.72-1.15)

Abbreviations: ciKMC, community-initiated kangaroo mother care; RR, relative risk.

^a^ Adjusted for birth order categories and accounting for household clustering.

^b^ The primary outcome is moderate-to-severe postpartum depressive symptoms, which are defined as a Patient Health Questionnaire–9 score of 10 or higher.

^c^ The adjusted model had an Akaike information criteria value of 0.73 and a bayesian information criteria value of −12352.16. The deviance statistic (*G*^2^) is calculated to be 1322.33, with 1821 *df*. The *G*^2^ corresponds to [−2 × (maximized log likelihood of the model of interest)] = [−2 × (−661.17)] and the *P* value referred to the deviance test is >.99, suggesting that the model is valid.^36^

^d^ Any depressive symptoms are defined as a score of 5 or higher on the Patient Health Questionnaire–9. A total of 159 of 852 mothers (18.7%) in the control group and 189 of 974 mothers (19.4%) in the ciKMC group had mild depressive symptoms.

In the subgroup of mothers with preterm births, the RR of moderate-to-severe postpartum depressive symptoms in the ciKMC group was 0.71 (95% CI, 0.52 to 0.96) vs 0.86 (95% CI, 0.56 to 1.34) among mothers with full-term infants. Preterm birth had a relative excess risk due to interaction with ciKMC for the study outcome of 0.19 (95% CI, −0.20 to 0.58).

Salivary cortisol measurements were done in 92.4% of the mothers (508 of 550 mothers) (Figure 1). The median (interquartile range) day-28 maternal salivary cortisol concentrations in both the ciKMC group and control group were similar before and after breastfeeding (Table 3).

**Table 3.** Effect of ciKMC on Maternal Salivary Cortisol Levels Among Mothers at the End of The Neonatal Period^a^

Time point	Salivary cortisol levels, median (IQR), μg/dL	
	Control (n = 265)	ciKMC (n = 243)
Before breastfeeding	0.22 (0.16-0.31)	0.22 (0.17-0.29)^b^
After breastfeeding	0.18 (0.14-0.26)	0.19 (0.13-0.25)^b^

Abbreviations: ciKMC, community-initiated kangaroo mother care; IQR, interquartile range.

SI conversion factor: To convert cortisol to nanomoles per liter, multiply by 27.588.

^a^ For estimation of salivary cortisol levels, 550 mothers were enrolled of whom we were able to collect salivary specimens from 508 (92.4%) mothers at day 28, before and after breastfeeding.

^b^ Wilcoxon rank-sum test did not suggest any difference.

## Discussion

We found a 25% lower risk of moderate-to-severe postpartum depressive symptoms among mothers in the ciKMC group than among those in the control group. The RR reduction was somewhat higher among mothers of preterm infants. Our analysis indicated that ciKMC practice for approximately 36 stable LBW infant-mother dyads (ie, the number needed to treat) would prevent 1 case of moderate-to-severe postpartum depressive symptoms. We found no difference in the day-28 maternal salivary cortisol concentrations between the study groups.

Our study was done as a substudy within the primary ciKMC efficacy trial.^16^ Selection and follow-up biases are not likely because of effective randomization, allocation concealment, and very low and balanced attrition. Nonetheless, we cannot rule out the possibility of recall bias. However, in the intervention group, information on the duration of SSC captured by the outcome assessment team based on mothers’ history was similar to that obtained by the intervention delivery team during their scheduled visits, suggesting a low likelihood of such bias.^16^ To minimize the possibility of conscious underreporting of symptoms, the outcome assessment team was extensively trained to conduct PHQ-9 assessment based on a comprehensive interview process in a comfortable home environment and not by asking the questions directly. Despite having an independent outcome assessment team, it might have been possible to guess the group allocation for those who practiced KMC beyond the 28-day period during PHQ-9 interview. However, such a possibility was small because only approximately 2% of mothers continued SSC beyond the 28-day period. Given the low risk of bias, the results are likely to be reliable and representative of the target population, suggesting that ciKMC can substantially reduce the risk of moderate-to-severe maternal postpartum depressive symptoms.

To the best of our knowledge, this is the first randomized clinical trial to estimate the effect of KMC on maternal depressive symptoms, and the findings substantiate those of previous reports from less rigorous study designs. In a quasi-experimental study in Canada,^20^ depressive symptoms measured by the Edinburgh Postpartum Depressive Scale scores at 1 week and 1 month postpartum were lower among 30 mothers who practiced SSC compared with 60 mothers who did not. Another quasi-experimental study^23^ of 50 Iranian mothers demonstrated that practice of KMC for 180 minutes daily for a week compared with when infants were cared for in incubators was associated with improved mean mental health scores assessed by the General Health Questionnaire. An observational study^21^ in Portugal among 177 low-income mothers with preterm infants showed that the proportion of women with postpartum depression assessed by the Postpartum Depression Screening Scale decreased from 37% after delivery to 17% at hospital discharge with KMC practice. Our trial provides valid evidence of the efficacy of KMC on the risk of postpartum depression in mothers with LBW infants and substantiates the observations from the earlier studies.

KMC may lessen depressive feelings by empowering women in their mothering role and improving infant-mother bonding.^19,22,37^ The substantial effect of ciKMC that we observed on postpartum depressive symptoms is likely to be a result of a multitude of complex psychological mechanisms, potentially supported and intensified by enhanced oxytocin release as a result of SSC and exclusive breastfeeding.^17,38^ Because of its clinical implications, we chose moderate-to-severe postpartum depressive symptoms as the primary outcome instead of PHQ-9 score. Women with PHQ-9 scores of 10 or higher have a high probability of major clinical depression that needs clinical attention, whereas the milder symptoms are often self-limiting.^28^ In this context, our findings that ciKMC may reduce the risk of moderate-to-severe postpartum depressive symptoms are pertinent. The potentially greater benefit of ciKMC in mothers with preterm infants in reducing moderate-to-severe postpartum depressive symptoms is relevant given their higher risk of postpartum depression.^11^ However, we acknowledge the limitations of our subgroup analysis, in that we did not stratify our randomization on whether the birth was preterm and because the statistical precision of the interaction was moderate.

Cortisol is a hormone produced by the activation of hypothalamic-pituitary-adrenocortical axis in response to physiological stress.^20,39^ Salivary cortisol is widely used to capture short-term fluctuations in physiological stress.^40^ The observed null effect of KMC on day-28 maternal salivary cortisol is similar to that of a quasi-experimental study in Canada,^20^ where the mean cortisol concentrations among intervention and control mothers at the end of neonatal period were 0.23 and 0.24 μg/dL, respectively. These findings suggest a possible dissociation between acute stress and postpartum depression, as far as the effect of ciKMC is concerned. Further research on the effect of KMC on acute and chronic stress (using markers such as hair cortisol^40,41^) may be warranted.

### Limitations

Although this is a large randomized clinical trial where almost all baseline characteristics were well-balanced between the trial groups, a baseline PHQ-9 assessment would have been valuable. Our study population was limited to mothers with stable LBW infants weighing 1500 to 2500 g, and the findings may not be applicable to mothers with unstable or very-LBW (ie, <1500 g) infants. Neonatal survival programs would benefit from further research that also includes such infants.

## Conclusions

The findings of our study support ciKMC as an intervention to prevent maternal depressive symptoms in the early postpartum period. Research on long-term benefits of the intervention on maternal psychosocial health and child development would be helpful. The study findings together with previous literature^13,16,20,23^ suggest substantial benefits of home-based KMC for mothers, beyond improving health and survival of LBW infants. Developing a focused LBW program is identified as one of the important agendas of the Indian Government^42^ and maybe relevant in other low- and middle-income countries where a high proportion of infants are born with LBW. This evidence supports the integration of KMC into essential newborn care programs for LBW infant-mother dyads.

## Notes

Supplement 1. **Trial Protocol and Statistical Analysis Plan**

Supplement 2. **eTable 1.** Practice of Kangaroo Mother Care Intervention Components and Home Visits Among Study Participants **eTable 2.** Effect of ciKMC on Different Categories of Postpartum Depressive Symptoms Among Mothers at the End of the Neonatal Period **eTable 3.** Proportion of Mothers With Postpartum Depressive Symptoms in Control and ciKMC Arm Using Different PHQ-9 Cutoff Scores

Supplement 3. **Data Sharing Statement**
