# Supplementary material for: Effect of Community-Initiated Kangaroo Mother Care on Postpartum Depressive Symptoms and Stress Among Mothers of Low-Birth-Weight Infants: A Randomized Clinical Trial
Source: JAMA Netw Open. 2021 Apr 22;4(4):e216040. doi: 10.1001/jamanetworkopen.2021.6040 (PMC8063066; doi:10.1001/jamanetworkopen.2021.6040)
Supplement: Supplement 1. — Trial Protocol and Statistical Analysis Plan [file jamanetwopen-e216040-s001.pdf]

Project application Form for application for approval of research project by the Regional Committees for Medical and Health Research Ethics (REC)

Dokument-id: 1131098

Effekt av hjemmebasert "Kangaroo mother care" på spedbarns inntak av brystmelk, tarmbetennelse, og tegn på mødres fødselsdepresjon og stress

1. General information

1.1 Chief Investigator

Name	Halvor Sommerfelt
Degree	Dr. med.
Clinical skills	Turnustjeneste (internship)
Position	Professor-SIH
Main place of work	Universitetet i Bergen
Work address	Senter for internasjonal helse (SIH)
Zipcode	5020
City	Bergen
Phone:	55588575
Cellphone	40640882
Email address	halvor.sommerfelt@cih.uib.no

1.2 Research project title

Norwegian title	Effekt av hjemmebasert "Kangaroo mother care" på spedbarns inntak av brystmelk, tarmbetennelse, og tegn på mødres fødselsdepresjon og stress
Scientific title of the research project	Effect of community initiated kangaroo mother care in low birth weight infants on infant breast milk intake, gut inflammation, maternal depressive symptoms and stress

1.3 Institution responsible for the research

Institution	Contactperson	Position	Email address
1. University of Bergen	Halvor Sommerfelt	Professor	halvor.sommerfelt@uib.no
2. Society for Applied Studies	Nita Bhandari	Director	nita.bhandari@sas.org.in

1.4 Initiator

Who is the initiator of the research project?	Chief Investigator and/or institution responsible for research (Funds-based research)

1.5 Educational project

Is the project part of a course of education or PhD study?	Yes
Study	Pediatrics/Medicine
Level	Postgraduate

1.6 Research project members

Name	Position	Institution	Academic ranking	Project role
1. Bireshwar Sinha	Research scientist	Society for Applied Studies	MD	PhD student
2. Maharaj K. Bhan	National Science Professor	Indian Institute of Technology	MD	PhD supervisor
3. Nita Bhandari	Director	Society for Applied Studies	PhD	PhD supervisor
4. Rajiv Bahl	Coordinator	World Health Organization	MD, PhD	PhD supervisor
5. Halvor Sommerfelt	Professor	University of Bergen	MD, PhD	PhD supervisor

1.7 Project time frame

Research project start date	02.10.2017
Research project end date	30.06.2020

1.8 Public access

Will exemption from public access be requested in the application or its attachments?	No

1.9 Collaboration with other countries

Does the project involve any form of collaboration with other countries?	Yes

☒ Independent collection of data in another country

Country	Place
1. India	Haryana

1.10 Other projects that are relevant to the consideration

Are there any other projects that may be relevant to the consideration of the current project?	Yes

Name of the research project

Impact of Promoting Kangaroo Mother Care (KMC) for Low Birth Weight Infants

Reviewed by REC	Yes
After May 5.2009	
Reviewed by	REC west
REC Project number	2015/1486

2. Research project information

2.1 Research project summary

Project description

Kangaroo Mother Care promotes survival in low birth weight infants through mechanisms yet to be elucidated. A large trial in India estimated the effect of community initiated Kangaroo mother care (ciKMC) on neonatal mortality. Within the trial, PhD student Bireshwar Sinha assessed the impact of ciKMC on infant breast milk intake, maternal postpartum depressive symptoms, a biochemical marker of maternal stress, and infant gut inflammation. Infant breast milk intake is measured by a stable isotope method using salivary samples. Maternal depressive symptoms were identified using the Patient Health Questionnaire (PHQ-9) and salivary cortisol levels were measured to reflect

maternal stress. The primary Objectives of the PhD studies are to estimate the effect of ciKMC on: 1. infant milk intake 2. prevalence of moderate to severe postpartum depressive symptoms and salivary cortisol levels 3. Gut inflammation as reflected in levels of fecal neopterin, myeloperoxidase, alpha-1-antitrypsin.

2.2 Clinical Trial

Clinical Trial	Yes

2.2.1 Testing phase

Testing phase	III (therapeutic confirmation)
EudraCT number	No number, please see attached "EudraCTForm_ciKMCBiolMech20181226.pdf"

2.2.2 Study medication

The name of the study medication

No medication, the intervention aims at a behavioural change through promoting community-initiated Kangaroo mother care (ciKMC).

Please state the dosage(s) that will be used in the study

No medication, the intervention aims at a behavioural change through promoting ciKMC.

Please state the reason(s) for the choice of dosage(s) that will be used in the research. Your reasons should include both biomedical reasons as well as reference to earlier studies (if any)

No medication, the intervention aims at a behavioural change through promoting ciKMC. For this reason, we selected "No" under "**2.2.3 Previous studies with study medication**".

2.2.3 Previous studies with study medication

Has there been any previous research carried out on this (study) medication?	No

2.2.4 Interruption to well-regulated treatment

Will patients be deprived of any approved treatment in order to participate in the study?	No

2.2.5 Follow-up of patients after study completion

Will patients that have entered into the study receive information about the outcome of the study and	No

share any benefits that result from it, for example, access to interventions identified as benefit

2.2.6 Research design

Control group(s)

☒ No treatment

Explain use of placebo or no treatment

This is a preventive behavioural intervention and the low birth weight babies in the control arm received standard of care.

2.3 Research data

**2.3.1 Previously registered information**	No
**2.3.2 New health data**	Yes

Please specify which types of personal health data

Breast feeding initiation, exclusive breast feeding, KMC adoption; vital status, weight, length, infant illnesses, care-seeking, maternal depressive symptoms at 1 month of infant age.

**2.3.3 Human biological material**	Yes
New human biological material	Feces, Saliva
☒ The material will be stored in a new specific general research biobank	Yes

Name of the biobank	Name of the person in charge of the biobank
Clinical and Research Laboratories Society for Applied Studies (CRL SAS), Devli, New Delhi	Dr. Ananya Tupaki Sreepurna (MD, Ph.D.)

Will genetic examinations be made of the biological material?	No

2.4 Study population

2.4.1 Number of research participants strength calculation

With 95% confidence and 90% power and expecting approximately 10% attrition, a total sample size of:

- 550 mother-infant dyads (275 in each group), will be sufficient to detect a 0.3 SD, i.e. 60ml, increase in infant breast milk intake with 90% power (1SD = 200ml),
- 1950 mother-infant dyads (975 in each group) will be sufficient to detect a 30% relative reduction in the prevalence of moderate to severe postpartum depressive symptoms (PHQ 9 ≥10), assuming a baseline prevalence of 19%,
- 550 mothers will be sufficient to detect a 0.3 SD i.e. 1.5 ug/dl decrease in the mean saliva cortisol levels (biomarker of stress),
- 250 infants (125 in each group) will be sufficient to detect a 0.5 SD reduction in the levels of gut inflammatory markers.

2.4.2 Description of research participants/selection

☒ Patients/Clients

Please specify which patient(s)

Participants

Mothers and low birth weight infants (≥1500 g to ≤2250 g) enrolled within 72 hours of birth

- Identified at home by surveillance team, either born at home or discharged from hospital without KMC having been initiated.

Please state the reason(s) for the choice of patient group(s)

The selected participants (babies with birth weight ranging from 1500 to 2250 g) and their mothers represent the target group for the intervention.

☒ Minors

☒ Under 12 years of age

2.5 Research method

2.5.1 Data analysis method

☒ Statistical (quantitative) research methods

2.5.2 Data gathering method

☒ Other type of intervention

Specify

Promotion of, and support for lactation management and skin-to-skin care as soon as possible after birth by study staff in addition to routine visits by government health workers.

☒ Medical examination

Specify

Examination for mouth ulcers as these could possibly influence saliva collection

Recording of baby weight and length

Illness identification in baby

Questionnaire-based screening for maternal depressive symptoms at 1 month of baby age

☒ Questionnaire

2.6 Explanation of choice of data and method

Explain the professional and scientific reasons for the choice of data and method

Efficacy design, as this is the first study of community-initiated KMC in India.

3. Information, consent and data protection

3.1 Consent will be obtained

Consent will be obtained Yes

For which participants, information and, if necessary, samples will consent be obtained?

Written consent was obtained from the caretakers of infants with birth weight > 1500 and < 2250 g who participated in the study.

How will the participants be identified, contacted and recruited? Describe the recruitment process and specify any reason for deviating from written consent

Study workers were allotted pockets of population for surveillance. They followed up pregnant women, weighed newborns as soon as possible after birth, and reported all babies weighing ≤2250 g to the study coordinator. They obtained verbal consent from families for reporting the information on the birth weight. Families with babies weighing ≤2250 g were visited by the screening and enrolment team. The members of this team explained the study to the caregivers and in those willing, obtained written informed consent for screening. Infants were weighed and their length was measured. The baby was screened for inclusion and exclusion criteria.

Describe inclusion criteria

Inclusion criteria

- Low birth weight singleton babies (≥1500 to ≤2250 g) screened within 72 hrs and their mothers.

- Born at home

- Infants born in hospital, discharged and KMC not initiated in the hospital

Describe exclusion criteria

Exclusion criteria

- Birth weight <1500 g

- Twins and triplets

- Confirmed inability to feed

- Major congenital malformations

- Infant with breathing problems or abnormally inactive

- Mother does not intend to stay in the study area for the next 6 months

- Mother expired

- Mother not living with the baby

3.2 Consent has already been obtained

Consent has already been obtained	Yes

For which participants, information and any possible samples has consent already been obtained?

All participants consented for participation and specimen collection.

Which body approved the previous information material?

Ethics Review Committee of the Society for Applied studies.

Main study "Impact of Promoting Kangaroo Mother Care (KMC) for Low Birth Weight Infants" (2015/1486) approved by REC.

3.3 An application is made for exemption from the requirement to obtain consent

An application is made for exemption from the requirement to obtain consent	No

4. Reconciling the benefits and risks of the project

4.1 Advantages

Specify the physical, psychological, social and/or practical advantages/benefits/gains, now or in the future, for the individual patients/participants, groups of people, society and/or science.

The immediate gains for those receiving the intervention can be reduced mortality, improved breast feeding and breast milk secretion and intake, improved immunity and reduced risk of gut inflammation, reduced risk of maternal stress and depression, and improved infant growth.

Knowledge about the biological mechanisms which may explain the impact of community-initiated KMC can be used to support the implementation of the interventions in similar settings.

4.2 Disadvantages

Specify the physical, psychological, social and/or practical risks/harm/discomfort/strain/inconvenience, now or in the future, for the individual patients/participants, groups of people, society and/or the environment.

The visits from our workers took up to 3 hours each time so a few woman became tired but none were fatigued. There were several visits in the home but less than 10 women, most of them in the control arm of the trial, seemed inconvenienced by our interaction with them. All women were comfortable after the team explained the importance and relevance of our examinations.

4.3 Measures

Explain any special measures to care for and protect patients/participants in the research project and to limit the potential risks/inconvenience

Infants who weighed <1500 g, were unable to feed, had breathing problems, were abnormally inactive or excluded for any other reasons, e.g. had major congenital malformations, were referred for care in a hospital. The research team facilitated referral through government health workers who were incentivized to effectuate referral. Problems faced by the mother during the intervention were identified, and mothers helped in resolving these issues using a Counselling Guide. If the mother reported any illness in the infant, referral was facilitated through government health workers.

To reduce the risk of aspiration during saliva specimen collection from the infants, a plastic forceps with “teeth” were used to hold the absorbent swabs. No event of aspiration or even swallowing of the swabs occurred during the trial.

4.4 Balanced justification of risks and benefits

Why is it justifiable to carry out the project? Provide a reconciliation of the advantages and disadvantages of the research project and specify the reasons

The potential benefits of the project clearly outweigh the inconveniences for the participants.

5. Security, interests and publishing

5.1 Identifiable information

In what form should identifiable information and samples be used in the project?

☒ Non-identifiable with use of a connection key

Provide information about how the connection key is stored and who has access to it

The information that we collect for this research project is kept private and confidential. Each participant was given an identification number for the study and only the researchers directly involved in the study have access to the key which links this identification number to the information collected during the study. This linkage is stored safely under lock and key.

5.2 Internal control and security

5.2.1 How will the identifiable information and samples be stored?

☒ Locked up storage

Please explain in detail the routines for storage and access to data

The information that we collect for this research project is kept private and confidential. Each participant was given an identification number for the study and only the researchers directly involved in the study have access to the key which links this identification number to the information collected during the study. This linkage is stored safely under lock and key.

☒ Connection key and data are stored separately

☒ Personal data (address, occupation, etc.) is stored and kept apart from other data

☒ Password protected storage

5.3 Insurance for research participants

☒ Insurance is not required/necessary

Please state the reason(s) why insurance will not be included for participants

No medicinal product/device is being tested in the trial.

5.4 Consideration by other authorities

The research project has been assessed/will be assessed by:

☒ Own institution

☒ Place of data collection

Which institution(s) or authority/authorities?

Society for Applied Studies, New Delhi, India.

☒ Authority/Authorities in another country

Which institution(s) or authority/authorities?

Ethics Review Committee of the Society for Applied Studies, New Delhi, India.

5.5 Interests

Source of finance

The study is funded by:

the Centre for Intervention Science in Maternal and Child Health (CISMAC) through Research Council of Norway (RCN) grant (223269)

Department of Science and Engineering Board, Department of Science and Technology, Government of India

Additional funding is soughtfrom the Thrasher Foundation.

Compensation to institution

None

Honorarium/fees for the Chief Investigator/Co-Researcher(s)

None

Compensation for participants of the research project

None, except for a very small gift item (worth some NOK 5) as a token of appreciation for the use of participants' time.

Please state any conflicts of interest (if any) for the research project's Chief Investigator or co-researchers

No conflict of interest.

5.6 Publication

Are there any restrictions preventing the disclosure and publication of the results from the research project?	No

Please explain how you intend to report and publicise the results from the research project

The results of the research will be published in international biomedical journal(s) with peer review. The findings will also be disseminated in international and national conferences and communicated to local, national (Indian) and international (WHO) health authorities.

5.7 Data processing after project conclusion

How will personal information be handled after the conclusion of the project?

Data will be stored under lock and key, and kept for at least five years after the conclusion of the project.

6. Attachments

#	Type	Filename	Registered date
1.	Investigators Brochure (IB)	InvestigationBrochure-ciKMC20190104.pdf	04.01.19
2.	Invitation to participate	KMC Substudy_English Consent Form Version 3.0 dated 09102017_PDF.pdf	27.12.18
3.	Consent given	Renewal of Approval_06MAR2018.pdf	27.12.18
4.	Consent given	SAS ERC_Letter of Approval following amendment_21_Dec 2017.pdf	27.12.18
5.	Questionnaire	PHD FORMS- QUESTIONNAIRES_PDF.pdf	27.12.18
6.	Research protocol	PhD proposalBiresh_27Dec2018_pdf.pdf	27.12.18
7.	EudraCT form	EudraCTForm_ciKMCBiolMech20181226.pdf	26.12.18
8.	Chief Investigator CV	CV_1 Page_Halvor Sommerfelt_Vacc20181004_1.pdf	26.12.18

7. Declaration of responsibility

I declare that the research project will be implemented
☒ according to current laws, regulations and guidelines
☒ in accordance with information provided in this application
☒ in accordance with any conditions for approval given by REC

Proposal for studies towards a PhD at the University of Bergen

Effect of community initiated Kangaroo mother care in low birth weight infants on infant breast milk intake, gut inflammation, maternal depressive symptoms and stress

Short title: Biological effects of c-KMC

PhD Student

Dr. Bireshwar Sinha, M.B.B.S., M.D., DNB
Scientist (Young Investigator)
Society for Applied Studies
New Delhi, India

Contents

Title of Thesis 1
List of Abbreviations 3
Scientific Environment / Supervisors 4
Abstract 5
List of planned articles (for the PhD) 6
Introduction: 7
Literature Review 8
Theoretical Framework 9
Hypotheses 12
Objectives 12
Primary Objectives (for the PhD thesis): 12
Secondary Objectives (for the PhD thesis): 13
Outcomes (for the PhD thesis) 13
Primary 13
Secondary 13
Relevance of the proposed research 14
Methods 15
Brief description of the main trial (in which this research will be embedded) 15
Description of the sub-study 16
Role and Responsibility of all Participating Institutions 22
Ethics 22
Sources of Funding 23
Time frame 23
References 24
Annexures 28
Responsibilities of Supervisors 34

List of Abbreviations

ANM	Auxiliary Nurse Midwife
ASHA	Accredited Social Health Worker
AWHONN	Association of Women's Health, Obstetric and Neonatal Nurses
CI	Confidence Interval
EBF	Exclusive Breastfeeding
KMC	Kangaroo Mother Care
LBW	Low Birth Weight
ELISA	Enzyme Linked Immunosorbent Assay
IFBAT	Infant Breast feeding Assessment Tool
IMNCI	Integrated Management of Neonatal and Childhood Illness
MD	Mean Difference
MoU	Memorandum of Understanding
MUAC	Mid Upper Arm Circumference
NHM	National Health Mission
OA Team	Outcome Ascertainment team
PSBI	Possible Serious Bacterial Infection
RNA	Ribonucleic acid
SAS	Society for Applied Studies
SCH	Stratum Corneum Hydration
SD	Standard Deviation
SSC	Skin to skin contact
SIgA	Secretory Immunoglobulin A
TEWL	Trans Epidermal Water Loss
THSTI	Translational Health Science and Technology Institute
WHO	World Health Organization

Scientific Environment / Supervisors

Dr. Halvor Sommerfelt MD, PhD<br>(Principal Supervisor)	Professor<br>Department of Global Public Health and Primary Care<br>Centre for International Health<br>University of Bergen, Norway<br>Email: Halvor.Sommerfelt@uib.no
Dr. Maharaj Kishan Bhan MD<br>(Internal Co-supervisor)	National Science Professor<br>Indian Institute of Technology, New Delhi, India<br>Former Secretary, Department of Biotechnology, India<br>Email: rajkbhan@gmail.com
Dr. Nita Bhandari MBBS, PhD<br>(External Co-supervisor)	Senior Scientist & Director<br>Centre for Health Research and Development, Society for Applied Studies<br>New Delhi, India<br>Email: nita.bhandari@gmail.com
Dr. Rajiv Bahl MD, PhD<br>(External Co-supervisor)	Coordinator<br>Maternal, Newborn, Child and Adolescent Health Research and Development<br>World Health Organization, Geneva<br>Switzerland<br>Email: bahlr@who.int

Abstract

Kangaroo Mother Care (KMC) promotes survival in low birth weight (LBW) infants born in health facilities. The substantial survival benefits indicate that KMC may operate through a multitude of mechanisms, not only by preventing hypothermia. These other mechanisms are yet to be clearly elucidated. The key pathways may include reduced exposure to common pathogens and thereby reducing the risk of infections, improving nutrition by increasing breast milk output through reduced maternal stress and therefore increased infant breast milk intake, modulation of gut inflammation and the establishment of beneficial gut microbiome. Elucidating the pathways through which KMC operates would contribute to the evidence base and may support its widespread implementation.

The opportunity is provided by a large ongoing trial in India, in which the effect of community initiated Kangaroo mother care on outcomes such as infant mortality, possible serious bacterial infection and linear growth are being measured. In addition within this intervention trial as a part of this PhD study, we propose to assess the impact of community initiated KMC on infant breast milk intake and breastfeeding performance, maternal postpartum depressive symptoms, stress and infant gut inflammation. Other outcomes (not necessarily a part of the PhD) of interest are infant skin barrier function, and gut microbial composition in low birth weight (LBW) infants.

Innovative scientific methods will be used for measurement of other outcomes. Infant breast milk intake (breast milk quantity) will be measured by a non-invasive stable isotope method (deuterium oxide technique) using infant and mother's salivary samples. This procedure allows accurate and non-invasive data collection and overcomes previous methodological issues associated with infant weighing to estimate infant milk intake. Maternal depressive symptoms will be identified and characterized using the Patient Health Questionnaire (PHQ-9), and salivary cortisol levels will be measured to reflect stress. Selected gut inflammatory markers (myeloperoxidase, Neopterin and alpha-1-antitrypsin) will be estimated. The infant microbiome will be evaluated by targeted metagenomic analysis, amplifying stool bacterial 16s RNA.

The goal of this mechanistic study is to add evidence and to elucidate some of the biological pathways by which community initiated KMC may produce beneficial health effects while promoting KMC as a part of essential newborn care in LBW infants.

List of planned articles (for the PhD)

1. The effect of community-initiated Kangaroo mother care (c-KMC) in low birth weight (LBW) infants on infant breast milk intake and breast feeding performance

2. Effect of c-KMC on maternal postpartum depressive symptoms and stress

3. The effect of KMC in LBW infants on gut inflammation and its association with linear growth

*Other possible articles from this research (not necessarily a part of the PhD)–see Annexure 5

Introduction:

Globally, Low birth weight (LBW) babies (<2500 grams) constitute 15% of all live births(1). India accounts for more than 40% of all LBW babies globally with 7.5 million babies (or 20% of the country's live births) being born with LBW(2). These babies have a reduced survival probability and contribute to 60-80% of the overall neonatal mortality(2). The key determinants underlying high mortality in these children are hypothesized to be infections and poor nutrition. Therefore, promotion of better nutrition and control of infections in these babies is essential to reduce neonatal mortality.

KMC is endorsed by the World Health Organization (WHO) for increasing the survival and well-being of LBW infants, particularly those born with a weight less than 2000 grams. The key components include early and prolonged skin-to-skin contact (SSC) between the mother and baby along with exclusive breast feeding(EBF) (3). The evidence for its mortality reduction is from hospital-based trials, mostly in developed countries (4). But the potential pathways through which KMC reduces mortality remain largely unclear. A large impact of neonatal mortality in LBW babies is plausible and may be mediated through several pathways driven through SSC; better mother-infant bonding, and protection conferred by breast milk. In addition to the documented effect of improved thermoregulation, the plausible pathways through which KMC operate may be reduced pathogen exposure and thereby prevention of infections, improvements in breast milk quantity and quality through reduced maternal stress, thereby promoting better nutrition and growth, early establishment of successful breastfeeding, better skin barrier, reduction in gut inflammation and establishment of a gut microbiome rich in *Bifidobacteria* and with fewer pathogenic bacteria. In a large ongoing trial, investigators of Society for Applied Studies, New Delhi, India, are examining the effect of c-KMC on neonatal and infant mortality, possible serious bacterial infection and linear growth. This PhD research aims to build in mechanistic sub-studies within this large trial to assess the effect of c-KMC on infant breast milk intake and breastfeeding performance; maternal postpartum depressive symptoms and stress; infant gut inflammation which are important biological outcomes related to growth promotion and infection prevention.

The knowledge gained from this PhD study on these important biological outcomes will be valuable to develop explanatory models to better understand the clinical impact of KMC on infant growth, infection reduction and survival in even though causality of the associations may

be difficult to establish. This PhD study is a pragmatic approach to link mechanistic science to the primary trial measuring clinical outcomes.

Literature Review

Hospital based KMC can result in a 40% relative reduction in post-KMC initiation neonatal mortality in LBW infants (4). However, there seems to be very limited evidence on the effect of KMC on biological pathways, particularly on whether it can affect infant breast milk intake, breastfeeding performance, maternal depressive symptoms or stress biology, gut inflammation and other outcomes such as - gut microbial composition, breast milk quality, and infant skin barrier function.

There is scarce evidence on the effect of c-KMC on the incidence of neonatal infections. However, in a recent Cochrane review (2016) of hospital-initiated KMC trials in developed countries, a 55% relative reduction in the occurrence of nosocomial infections or sepsis until discharge in LBW babies from the birth facilities was noted(4). In the same review, the findings from two small trials revealed a statistically significant increase in length gain per week (mean difference (MD) 0.29cm, 95%Confidence Interval (CI) 0.27; 0.31) at the latest follow up or at discharge. There were no data on later time points i.e. at 1, 3 or 6 months of life (4).

Currently, trial registries in India show that there are some ongoing studies on KMC. Most of these are hospital based trials which examine the effect of KMC on very LBW (<1500 g) babies, growth and survival of the babies and breastfeeding outcomes. International registries show that, the ongoing intervention trials are mostly hospital-based and examine the effects of KMC on hypothermia prevention, weight gain in neonates, breastfeeding, heart rate and other vital signs.

The only previous trial on c-KMC was done in Bangladesh, but it could not provide sufficient conclusive evidence to demonstrate benefits of KMC initiated at home(5). The trial had several methodological weaknesses, and the authors concluded that the extensive missing records for birth weight (~45%) and weak implementation of KMC in the study (only 47% mothers in the intervention group provided SSC ≥4 hours within the first 2 days of birth) rendered the study findings unreliable. Additional experimental studies are needed to assess the benefits of c-KMC. Investigators at the Society for Applied Studies, New Delhi are conducting a randomized controlled trial to examine impact of Kangaroo mother care initiated at home on neonatal and infant mortality, on the incidence of infections and on linear growth in 10500 infants weighing

between 1500 and 2250 g in Faridabad and Palwal districts of Haryana(6). The possible pathways by which KMC may promote survival or improve growth remain unexplored. Currently, there seems to be no other ongoing trial that examines the effect of c-KMC on linear growth or reduction in the incidence of possible serious bacterial infection or the underlying biological pathways.

Theoretical Framework

Hospital initiated KMC has been shown to lead to a 40% (95%CI 8%, 61%) relative reduction in the neonatal mortality of LBW infants. It was earlier perceived that the mortality benefit may be mainly related to hypothermia prevention(7). However, it is unlikely that this pathway alone can explain the large survival benefit and other important underlying mechanisms are likely to be operational. Soft evidence suggests the key pathways may be through promoting better nutrition and growth, reduced pathogen exposure and thereby reduction of the incidence of infections(8).

Growth retardation begins *in utero* and may continue until 2 years of age and beyond. The average length-for-age Z-score among newborns in developing countries is approximately -0.5 at birth and continues to decline to reach a nadir of -2.0 LAZ by 24 months of age. The period from conception to a child's second birthday has been identified as the most critical window of opportunity for interventions(9). Preliminary evidence from two small hospital based studies suggests that KMC initiated soon after birth may promote linear growth at latest follow up or at discharge; however there is no conclusive evidence (4). It is postulated that KMC may promote linear growth through a combined effect on: early establishment of successful breastfeeding, improvement in breast milk intake, reduction in maternal depression and stress, reduction of gut inflammation and establishment of a Bifidobacterium rich infant gut microbiome.

LBW infants are vulnerable to have more infections. Therefore, infection reduction is a key pathway through which KMC may improve survival in LBW infants (4, 10). It is possible that KMC may act to reduce infections through improvements in breast milk intake, breast milk anti microbial proteins, maturation of skin, reduction of gut inflammation and establishment of a beneficial Bifidobacterium rich infant gut microbiome modulating host responses against infection. Though causality of the relationships may be difficult to establish in the context of this trial, measurement of these important physiological outcomes will help to develop explanatory models to explain clinical impact on survival, growth or infection.

A) Infant breast milk intake: Breast milk is the primary source of nutrition in infants. Improved infant milk intake can prevent childhood undernutrition; confer protection against infections, promote growth and survival in LBW infants. In mammalian biology, SSC following birth may promote self-attachment of the infant to the mother's breast, leading to early breastfeeding initiation (11-15). The SSC component of KMC, through sensory stimuli such as touch, warmth and odour, acts as a powerful vagal stimulant and releases maternal oxytocin(16). Oxytocin decrease maternal anxiety, increases confidence, leading to improved attachment, better milk output, successful breastfeeding and thereby improved infant milk intake (17, 18). Though, this is theoretically plausible, hard evidence to support this hypothesis would be of immense value.

B) Maternal postpartum depressive symptoms and stress biology: Post-partum depression is known to be associated with several negative effects on infants, such as impeded growth, less mother-child interaction and poor breastfeeding. Post-partum depressive symptoms have been measured using the Patient Health Questionnaire 9 (PHQ-9). A score of ≥10 in the PHQ-9 signifies moderate to severe postpartum depressive symptoms(19) and is 91% specific compared to the DSM-IV criteria (gold standard) to diagnose major depression in the postpartum period(20).It is estimated that around 19-25% of Indian mothers suffer from have moderate to severe postpartum depressive symptoms(21-23). Preliminary evidence suggests that SSC during the neonatal period may prevent depressive symptoms and lower stress (cortisol levels) in the mothers and thereby improve breastfeeding performance and growth in infants (24-26). It would be clearly valuable to demonstrate the effect of KMC on maternal depressive symptoms and stress biomarker levels e.g. salivary cortisol, given the importance of the problem and its conceivable relationship with infant health outcomes.

C) Gut inflammation: Gut inflammation seems to be almost ubiquitous among young children in impoverished communities(27). This has been linked to subsequent linear growth deficits and is postulated to be one of the important barriers to the limited success of various growth promoting interventions(28). Repeated exposure to multiple enteric pathogens in LBW infants living in poor socioeconomic communities results to gut injury and a chronic, indolent inflammatory state of the gastrointestinal tract. This, in turn, causes protein and micronutrient malabsorption leading to a vicious cycle of malnutrition and poor growth(28, 29). However, it is still unclear whether gut inflammation is reversible and if so what possible interventions might work to reduce gut inflammation. New developments in this field suggest that surrogate fecal biomarkers of gut inflammation (Myeloperoxidase (MPO), Neopterin

(NEO) and Alpha 1 antitrypsin (AAT) may be used as a proxy measure and this can potentially predict subsequent linear growth faltering in infants (29). In KMC as the baby is placed in a protective environment in direct skin to skin contact with the mother soon after birth and is exclusively breastfed, the chances of repeated pathogen exposure and ingestion of microbes from contaminated sources are reduced. It is plausible that this may lead to a reduction in gut inflammation and gut injury. .

D) Effect on gut microbiome: Altered gut microbiota is known to be associated with infant undernutrition, impaired skeletal and somatic growth (30, 31). Undernourished children have been shown to have a relatively immature gut microbiota for their age(30, 31). Studies in animal models suggest a c*ausal relationsh*ip between microbiota and growth. When transferred to germ free mice, microbiota from undernourished children *was found to* impair the physical growth and that from well-nourished children prevented growth impairment and promoted somatic and skeletal growth in the ani*mals (32)*. It has been seen that the newborn gut is initially colonized by *Enterobacteria.* In infants who are almost exclusively *breastfed the m*icrobiome pattern changes by around 1 month of age and has been seen to comprise predominantly of the *Bifidobacterium* group which are milk oligosaccharide fermenters (31, 33). The establishment of a healthy gut microbiome with abundance of *Bifidobacterium* may modulate infant host responses and prevent infections(31).It is plausible that KMC may influence the establishment of a *Bifidobacterium* rich gut microbiome in the infants similar to that of the healthy exclusive breastfeeding babies through maternal transfer, better breastfeeding performance and reduced exposure to other bacteria. But, this has not been tested. Hence, the questions of whether KMC favors the establishment of a gut microbiome rich in Bifidobacteria that may promote growth and prevent infections beg answers.

E) Maturation of skin barrier function: In LBW infants, skin barrier functions are compromised due to the thinner and less developed stratum corneum layer, leading to increased risk of cutaneous infections (34). SSC may help to reduce the risk of infections by maturation of the LBW baby's skin (35, 36). A recent quasi experimental study indicated an increase in stratum corneum hydration in infants during SSC in all areas that were in direct contact with the mother(37). Though this study indicates that KMC may improve skin barrier function, more research is needed.

The conceptual framework in Annexure 1 shows the hypothesized pathways through which the effects of KMC may operate as per our literature review. In this PhD study we will evaluate the effect of KMC on improved infant breast milk intake, maternal depressive symptoms (measured by PHQ9) and stress (measured by cortisol levels), gut inflammation and breastfeeding performance. This study will be embedded within the ongoing randomized controlled trial in Haryana, where the effect of cKMC on clinical outcomes viz. mortality, linear growth and infections are being studied(6).This is a pragmatic approach to link mechanistic science to the intervention trial. Measuring these physiological and biological outcomes will help to build explanatory models to explain the clinical impact of KMC on growth, infection or survival although it is recognized that causal relationships may be difficult to establish in this experimental setting.

The other outcomes of interest are gut microbial composition, breast milk antimicrobial proteins and infant skin barrier function, in the LBW infants which will be a part of this research work but not necessarily a part of the PhD.

Hypotheses

In LBW infants, c-KMC leads to:

1. Improved infant breast milk intake (at least 60 ml increase);
2. Substantial reduction in the prevalence of postpartum moderate to severe depressive symptoms (prevalence ratio ≤0.7) and substantial reduction in salivary cortisol levels (reduction of at least 0.3 SD in mean values) as a marker of maternal stress.
3. Reduction of gut inflammatory markers -Fecal Neopterin, Myeloperoxidase, alpha1antitrypsin (reduction of at least 0.5 SD in mean values)

Objectives

Primary Objectives (for the PhD thesis):

To estimate the effect of community initiated Kangaroo mother care during the neonatal period in LBW infants on:

1. Infant breast milk intake during neonatal period

2. Prevalence of moderate to severe postpartum depressive symptoms (Patient Health Questionnaire-9 score ≥10) and stress (measured by salivary cortisol levels) at the end of the neonatal period
3. Gut inflammation as reflected in levels of fecal neopterin, myeloperoxidase, alpha1antitrypsinat the end of the neonatal period.

Secondary Objectives:

1. To study the effect of c-KMC on prevalence of mild depressive symptoms (PHQ 9 score 5-9) and suicidal ideation in mothers in postpartum period.
2. To study the effect of c-KMC in LBW infants during the neonatal period on successful breastfeeding performance (measured by IBFAT score[1]), complete lactation failure[2], perceived breast milk insufficiency[3] and use of breast milk substitutes;
3. Association of Gut inflammation (Fecal Neopterin, Myeloperoxidase, alpha1antitrypsin) in neonatal period with linear growth in the first 6 months of life.

Outcomes

Primary

1. Infant breast milk intake during the neonatal period
2. Moderate to severe maternal postpartum depressive symptoms (Patient Health Questionnaire-9 score ≥10) and salivary cortisol levels at the end of neonatal period
3. Gut inflammatory markers: Fecal Neopterin, Myeloperoxidase, α1-antitrypsin at baseline and at the end of neonatal period

Secondary

1. Mild depressive symptoms (PHQ-9 score 5-9), mean PHQ-9 scores, mothers reporting suicidal ideation in postpartum period
2. Complete lactation failure(38), Perceived breast milk insufficiency(39) and Use of breast milk substitutes during the neonatal period (enrolment to end of neonatal period).

---

[1]IFBAT-based on 4 parameters i.e. readiness to feed, rooting, latch on and sucking pattern
[2]Complete Lactation Failure- Total absence of milk flow or secretion of only a few drops of milk following regular suckling for a period of at least 7 consecutive days
[3] If the mother perceives that she does not have enough milk then it is defined as perceived breast milk insufficiency as per WHO 2009 IYCF guidelines.

3. Successful establishment of breastfeeding (measured through infant breastfeeding assessment tool –IFBAT) at the end of neonatal period
4. Attained LAZ scores at 1 month, 3 months and 6 months of age; linear growth velocity between birth to 3 months and 3 to 6 months of age

Annexure 4 provides detailed time points of measurement of each outcome.
Annexure 5 provides details on other objectives and outcomes of this research (not essentially a part of the PhD)

Relevance of the proposed research

KMC is a novel intervention for improving survival and well-being of LBW infants. Scaling up KMC even in hospitals is a challenge because many pediatricians, at least in India, are still unclear about the need for it wherever incubators are available. Knowledge on how this intervention works is lacking. The perception that SSC early in life may have profound biological and clinical effects beyond thermoregulation are not common and more research is needed. As most of the KMC trials have not been done in India, pediatricians and/ or policy makers in our country question the importance of this intervention in the Indian context. Currently, despite the high proportion of babies that are born with LBW, in most of the facilities in India, KMC is not being implemented; also LBW babies born at home miss out on this intervention which has the potential to improve their survival, growth and reduce their morbidity. To demonstrate how KMC might work to reduce mortality and morbidity in LBW infants it is important to understand the underlying biological mechanisms.

This PhD work will provide critical new knowledge on the possible underlying biological mechanisms behind any beneficial effects of c-KMC, i.e. infant breast milk intake, maternal stress (measured by cortisol levels) and depressive symptoms, breastfeeding performance and gut inflammation. Additionally, we will explore the effect of KMC on infant skin barrier function, gut microbial composition in the LBW infants. We hope that, through better understanding of the biological pathways, KMC and, if found effective, c-KMC may be better accepted to the pediatricians and policy makers and thereby be more effectively strongly promoted at all levels of the health system.

Methods

Brief description of the main trial (in which this research will be embedded)

To answer the objectives of this research, a sub-study will be embedded in the large ongoing individually randomized controlled trial entitled *"Impact of Community-initiated Kangaroo Mother Care on Survival of Low Birth Weight Infants"* (www.clinicaltrials.gov #NCT02653534) where 10,500 LBW infants will be enrolled to examine the impact of community initiated KMC on neonatal and infant mortality(6).The identification, screening and enrolment of mother-infant dyads for this sub-study will be embedded within the main trial.

Setting: The trial is being conducted in the rural and semi-urban areas of districts of Faridabad and Palwal in the state of Haryana (India), with a population of ~2 million, annual birth rate of 25.6/1000 population with around 20% of the babies being born with LBW.

Population: The study population comprises of mother infant dyads with babies weighing ≥1500 g to ≤2250 g enrolled within 3 days of birth identified by the study surveillance teams, either born at home or discharged from hospital and for whom KMC was not initiated in the hospital.

Inclusion and exclusion criteria: Infants unable to feed, with breathing problems, major congenital malformations or less active on the day of visit are referred to hospitals. Those intending to move away over the next 6 months or refuse participation are also excluded.

Screening, enrolment and randomization: In the main trial, pregnant women are being identified and followed up, their babies are weighed at birth and enrolled if eligible by study workers. Additionally, infants who are ill or whose weight is <1800gare counseled to visit health facilities as mandated by government.However contact is maintained with such families and they are screened for enrolment when discharged from hospital if still within the 3 day enrolment window. Eligible infants are randomized using a randomization list prepared by an offsite statistician from WHO not otherwise involved with the trial. Allocation of subject IDs is done using serially numbered opaque sealed envelopes. At baseline, following consent, a questionnaire is administered to obtain sociodemographic details and child anthropometry is done.

Intervention: The intervention group will receive KMC, i.e. promotion of and support for SSC and EBF as soon as possible after birth by study Auxiliary Nurse Midwives (ANMs) supported by study Accredited Social Health Activists (ASHAs) in addition to the routine postnatal visits by government health workers. The study visits are made daily for the first 3 days, on days 5 and 7,

twice in the second week and once each in weeks 3 and 4. Study ANMs and ASHAs visit together on the first 3 days. Visits will continue till 28 days or till the baby wriggles out. Information on the duration of SSC per day and number of breastfeeds over the last 24 hours will be captured. For any illness, referral will be facilitated through government ASHAs.The babies in the control arm of the trial receive routine postnatal visits by government health workers as implemented through the health system.

Description of the PhD sub-study

Sample sizes for the sub-study (Vide Annexure 3):

With 95% confidence and 90% power and expecting approximately 10% attrition, a total sample size of:

- 550 mother-infant dyads (275 in each group), will be sufficient to detect a 0.3 SD, i.e. 60ml, increase in infant breast milk intake with 90% power (1SD = 200ml),
- 1950 mother-infant dyads (975 in each group) will be sufficient to detect a 30% relative reduction in the prevalence of moderate to severe postpartum depressive symptoms (PHQ 9 ≥10), assuming a baseline prevalence of 19%,
- 550 mothers will be sufficient to detect a 0.3 SD i.e. 1.5 ug/dl decrease in the mean saliva cortisol levels (biomarker of stress),
- 250 infants (125 in each group) will be sufficient to detect a 0.5 SD reduction in the levels of gut inflammatory markers.

Therefore, the overall sample size will be 1950 mother infant dyads in whom the prevalence of postpartum depressive symptoms will be assessed using PHQ 9. Infant breast milk intake, and salivary cortisol (biomarker of stress) will be assessed in a sub-sample of 550 subjects; Infant gut inflammatory markers will be assessed in 250 subjects. Information on the linear growth-related outcomes and possible serious bacterial infection will be available for all these study subjects. The secondary outcomes related to breastfeeding i.e. lactation failure, perceived milk insufficiency, breastfeeding performance will be measured in the sub-sample of 550 subjects.

Inclusion and Exclusion: Singleton Low birth weight babies (≥1500 to ≤2250 g) aged >72 hours to 7 days who are either born at home or hospital but KMC not initiated in the hospital and are already a part of the trial titled “Effect of Community Initiated Kangaroo mother care on babies with Low Birth Weight” will be included in this sub-study.

In the sub-study, we will exclude infants whose mother expired or is not living with the baby for any reason. Also for the subsample of the 550 mothers where infant breast milk intake will be assessed, twins or triplets (~5% of the total LBW births) will be excluded because of the difficulties involved in accurately assessing breast milk output in mothers with twins/ triplets using the deuterium oxide method.

Enrolment in the sub-study and collection of baseline data: Out of all the LBW infants enrolled in the main trial, a sub-sample of the LBW infants aged >72 hours to 7 days and their mothers will be included in this sub-study. Among the subjects enrolled in the main trial in a day, 4-6 subjects will be selected randomly per day and approached by the supervisor on Day 4 for obtaining additional consent for the sub-study. In case of non-consent, the subsequent mother-infant dyad will be approached. Data will be recorded to keep track of mothers who consented and did not provide consent. In these women, additional samples or information will be collected for the sub-study. Baseline information will be collected for the sub-study to describe the study sample and to adjust for any confounding. These variables include sociodemographic details (age, years of schooling of the mother, wealth index, total family income, family type, religion and working status of mother), reported gestational age, birth order, place and mode of delivery, birth weight of the baby, previous breastfeeding experience, maternal dietary habits, iron and calcium tablet intake, history of maternal post-partum illnesses (fever). Maternal anthropometry (height and weight) will also be done at enrolment to ascertain baseline nutritional status.

Informed Consent: Written individual informed consent for this sub-study will be obtained from the caregivers at enrolment, from whom additional samples and information will be collected. The informed consent form for the sub-study has been translated into simple Hindi language that can be easily read and understood. Thepurpose of the study, procedures to be performed, samples to be collected and stored will be explained in detail. In those who are unable to read, the worker who obtains consent will read out the consent form. The mothers' understanding of the information will be confirmed. In those who are unable to sign, a thumb imprint will be taken which will be witnessed (counter signed) by an impartial literate witness. Only the eligible LBW babies, whose parents provide informed consent will be enrolled for this study.

Outcome ascertainment: Outcomes specific to the sub-study will be measured by a independent team trained in the study related measurements and laboratory procedures in the study subjects enrolled through home visitations. There will be a total of 4-5 such teams. Each team comprises

2 members i.e. a supervisor trained in biological sample collection, (including saliva and stool), and a field worker to help during sample collection and interviewing. A Coordinator will ensure and coordinate all sample collection, as well as storage and transport of samples. Training is provided to the Supervisors for the interviews with the PHQ 9(19) and breastfeeding assessment questionnaire(40).

Anthropometry measurements (length and weight) infection ascertainment will be done when the babies are 1, 3 and 6 months of age as a part of the main intervention trial.

Non-invasive stable isotope methodology (deuterium oxide^2H_2O technique) will be used to assess infant milk intake. This allows for accurate and non-invasive data acquisition and overcomes previous methodological issues associated with infant weighing (41, 42). Infant breast milk intake measurement will be done using salivary samples from the mother and baby collected sequentially over a two-week period starting from the day of enrolment to get correct estimates as by this time milk production usually reaches optimal levels (43). Lactating women will be given an oral drink containing 30g of ^{2}H2O at enrolment. Saliva samples will be collected at baseline (day of enrolment), day1, 3, 7, 13 and 14 after enrolment using sterile cotton swabs. Saliva samples will be stored in a cool box by trained field workers and transported to the storage facility within 6-8 hours. This will be stored in the-20°Cfreezersin the Central Research Laboratories - Society for Applied Studies (CRL-SAS), Devli until analysis as per International Atomic Energy Agency (IAEA) guidelines(41). The samples will be shippedin batches on dry ice to St. John's Research Institute, Bangalore, for analysis within 6-12 months of collection. The appearance and subsequent disappearance of ^{2}H2O will be analyzed by enrichment of ^{2}H2Oin mother and baby saliva samples using the Fourier transform infrared (FITR) spectophotometry. The infant breast-milk intake will be expressed as the area under the curve over this 14 day period; this will help us to take into account the day-to-day variability of the breast milk intake.

Breastfeeding performance will be assessed using the validated IBFAT breastfeeding assessment tool (40) which is based on 4 parameters i.e. readiness to feed, rooting, fixing (latch on) and sucking. Higher score denotes better breastfeeding. Lactation failure, perceived breast milk insufficiency and use of breast milk substitutes during the neonatal period (enrolment to day 28) will be assessed by interviewing mothers with a semi-structured questionnaire on day 29adapted from WHO/UNICEF resource material on breastfeeding counseling and UNICEF breastfeeding assessment tools (https://www.unicef.org.uk/babyfriendly/baby-friendly-

resources/guidance-for-health-professionals/tools-and-forms-for-health-professionals/breastfeeding-assessment-tools/).

Assessment of depressive symptoms in mothers will be done at the end of the neonatal period i.e. day 28 of life, by the trained OM team using the validated PHQ-9 questionnaire which is also pre-tested (19). For maternal salivary cortisol determination which is being used as an indicator of stress, fasting salivary samples will be collected by placing cotton swab in the oral cavity of the mother in the morning around 7-8 am when the baby is 28 days old. The morning salivary sample at 7- 8 am is seen to correlate with the entire area under curve of the awakening response of cortisol and even a single sample per participant could assess whether a mother had lower or higher concentration of cortisol (44). As the cortisol levels may also vary in relation to breastfeeding (45), we will collect two salivary samples from each mother and baby in the morning (around 8 am) before and after breastfeeding when the baby is 28days old. The time of salivary sample collection and previous breastfeeding will be noted. The samples will be aliquoted, labeled and transported in cold boxes to the storage facility at CRL-SAS, Devli within around 8 hours where it will be stored in -20 ^{0}C in freezers until further analysis. Analysis will be done in CRL-SAS using quantitative ELISAs. The kits will be validated in our lab prior to testing of study samples. We also plan to collect saliva samples from the infant at day 28 as an exploratory exercise to study relationship of infant stress with maternal stress and its association with milk intake, though it is not a part of our primary objectives.

For stool collection, mothers will be provided with a cold box with 4 ice packs andsterile stool container at the day of enrolment and on Day 28 by the study workers. To ensure the cold chain the family will be asked to keep 2 gel/ice packs in the cold box and 2 in a freezer in their home (for families who have a refrigerator) or in a nearby home or shop. They will be asked to change the gel packs in the ice box after about 12 hrs. The worker will educate the mother about stool collection using the spoon following aseptic precautions in the sterile stool vial. Around 5-10g of stool sample (1 to 2 spoon) will be collected. After stool collection in the vial, the vial is to be placed in the cold box with all the 4 ice packs. The caregiver/ mother will be instructed to collect stool samples only when the infant passes stool between 6am to 3pm (noon) because of operational feasibility and prompt transportation of the samples on the same day. The mother will be provided with a phone number to inform the study team as soon as possible after stool collection. Alternatively, the field worker will also call the family intermittently to get confirmation on stool collection. A maximum wait time of 3 working days will be allowed to get stool specimen

of each infant at each time point. After telephonic confirmation of stool collection, the field worker will visit and collect the stool specimen with the cold box from home. Collected stool samples will be transported from the home to the field office at Palwal within 3-4 hours where it will be stored in the refrigerator (2-8 degrees). At the end of each day (i.e. 8-10 hours of sample collection) the samples will be transported in cold boxes to the storage lab where the samples will be stored at -80°C till further analysis. Analysis of the gut biomarkers will be done using commercially available ELISA kits at Clinical and Research Laboratories, Devli, New Delhi. A fully automated ELISA Evolis Twin Plus (Biorad) system will be used. All the kits will be validated in our lab prior to testing of study samples. Gut microbiome analysis(not necessarily a part of the PhD), will be done inCenter for Human Microbial Ecology (CHME) laboratory in Translational Health Science and Technology Institute (http://www.thsti.res.in/chme/about_chme.php).

Though not necessarily a part of the PhD objectives, breast milk will be collected on day 14 after enrolment (i.e. in the third week of life as the infant will be enrolled at Day 4-7) during daytime in 250 mothers to assess breast milk oxytocin levels and breast milk anti-microbial proteins using standard quantitative ELISA kits (46) which have been validated per standard protocols (47, 48). This is because the breast milk protein levels reach a fairly steady state around the 2nd week of life and there is little variation thereafter (43, 49, 50).The collected breast milk will be transported in cold boxes (2-8^{0} C) to the storage facility (CRL-SAS Devli) within 8-10 hours will be aliquoted in smaller vials and stored at -80^{0}C until further analysis. An independent Data Safety Monitoring board will look at the SDs of the breast milk protein analytes and breast milk oxytocin levels after 100 assays (50 in each group) and based on that we will have a provision to decide the sample size. Assessment of skin maturation in infants will be done at day 28 of life using the “AWHONN Neonatal skin score” tool (51). This scoring system is based on 3 parameters, i.e. dryness, erythema and breakdown of skin with scores ranging from 1-3 in each category. Lower score suggests higher skin maturation.

Note: Though the breast milk samples and stool samples are being collected and will be stored at -80^{0}C,the complete analysis for the breast milk (oxytocin and antimicrobial proteins) and stool samples (gut microbiome) will be done if and when additional resources are available.

Training and standardization: Prior to study initiation, different categories of staff will be trained in Good clinical/laboratory practices, procedures, measurements and individual responsibilities.

Training will also be given on assessment of breastfeeding performance, postpartum depressive symptoms using different tools i.e. IBFAT questionnaire and PHQ-9, respectively. The objectives and full protocol should be made available only to the staff that will not handle the mother-baby dyads directly. Lab personnel will be completely blinded to whether the specimens come from a cKMC or a control baby/mother. Study workers will also be trained in safe and aseptic handling of biological samples and relevant laboratory operations. All measurements/assays will be appropriately standardized and verified.

Quality control activities: A predefined proportion (1-2%) of enrolments and home visits by outcome ascertainment team will be observed by an independent team.

Data management and custody: The Data Management Centre (DMC) will be set up in the field office. Data will be collected on paper or electronic forms. Double data entry will be done by a data entry operator. Range and logical checks will be incorporated to ensure correct data entry. Data will be transferred to a local server at the DMC. Checks across forms and logical error checks will be run at the DMC. Queries generated will be given to study team for resolution within 48 h and corrections incorporated promptly.

The investigator, data management team will have custody over the data. All the computers and screens will be password protected. All personnel in the data management centre will be given user access authorization.

Data analysis: Data analysis will be carried out using STATA version11.2 or a later version (Stata Corp, College Station, TX, US). All analyses will be conducted on an intention-to-treat basis. Socioeconomic and demographic characteristics of the intervention and control groups will be examined for group comparability. Any large differences will be controlled for during data analysis. The frequencies of the study variables will be examined to assess the distribution of data. If the data are not normally distributed, decisions about the need for data transformation and the appropriateness of statistical tests will be made.

For quantitative outcomes, means and standard deviations and mean differences will be reported. Unpaired t-tests (in normal data) or Mann-Whitney test (for data with very skewed distributions) will be done to examine the difference between intervention and control groups for breast milk intake, maternal PHQ-9 score, IBFAT score, salivary cortisol levels and gut inflammatory markers. To estimate the effect of KMC on different quantitative outcomes e.g.

infant breast milk intake, gut inflammation, multivariable linear regression models will be used. Covariates identified as potential confounders will be included in the regression models for adjustment. Regression analysis will also be done to examine the effect of infant milk intake, postpartum depressive symptoms and gut inflammation on linear growth velocity (birth to 3 months and 3 to 6 months) and LAZ scores of the LBW infants. To assess dose-response relationships between duration of KMC and volume of breast milk output generalized additive models shall be used. We may also use Instrumental Variable analyses to study associations between actual intensity of SSC and Breast feeding and the outcomes of the proposed studies.

For qualitative outcomes e.g. mothers with PHQ9 score ≥10, lactation failure, perceived milk insufficiency, proportions will be reported. If necessary to adjust for any baseline differences, univariable and multivariable logistic regression analysis or analyses with generalized linear models of the binomial family with a log link shall be done to estimate the impact of KMC on these outcomes. Potential interaction between cKMC and all other variables on the outcome measures will be examined by including interaction terms in the multivariable regression models, after identifying subgroup differences by simple stratified analyses. In order to quantify biologic interactions(52) we will estimate the relative excess risk due to interaction(RERI) with its 95% CI(53, 54).

Role and Responsibility of all Participating Institutions

The partner Institutes are St. John's Medical College, Bangalore, which is one of the leaders in Nutrition Biochemistry in India and THSTI, which is an innovative institute leading research in Biotechnology. Dr. Anura Kurpad of St. Johns Medical College who will be a co-investigator of this research project, will help in training study team for ^2H_2O administration, saliva collection techniques and Lab analysis of Saliva samples for estimating Infant milk intake (breast milk output).Dr. Bhabatosh Das of THSTI who will be another co-investigator in this project will help in Lab analysis for Gut microbiome at THSTI lab.

Ethics

Ethics clearances have been obtained from the institutional Ethics Committee of the Society for Applied Studies for this sub study. The ethical approval will be provided to the Regional Committees for Medical and Health related Ethics (https://www.etikkom.no/rek) in Norway for information and additional comments (if any). We have a Memorandum of Understanding (MoU)

with National Health Mission (NHM) in Haryana and approval to do community based studies in partnership with the local government.

Sources of Funding

The study (PhD proposal) is partly supported (500,000 NOK) by the Centre for Intervention Science in Maternal and Child Health (CISMAC; project number 223269), which is funded by the Research Council of Norway through its Centres of Excellence scheme and the University of Bergen (UiB), Norway. This support will be used for analysis of infant breast milk intake using deuterium oxide method.

The research proposal has also been submitted to Department of Biotechnology, Government of India for additional funds required for the other objectives. A decision is awaited. The Society for Applied Studies, New Delhi (SAS) will contribute by enabling the PhD objectives to be pursued within the context of the trial and any shortfall in funds to complete the primary objectives for the PhD will be supported by SAS through institutional overheads.

Time frame

S/N	Activity	Timelines
1	Recruitment of staff, training and standardization of study workers, development of study instruments	1 month
2	Enrolment of subjects with ongoing biological sample collection	8 months
3	Follow up and completion of sample collection, anthropometry measurements and lab analysis of samples	6 months
4	Data cleaning and analysis	6 months
5	Paper writing (3 papers)	9 months
6	**Total duration**	**30 months**

References

1. Care of the preterm and/or low-birth-weight newborn [Internet]. World Health Organization. 2017 [cited 20 Dec 2016]. Available from: http://www.who.int/maternal_child_adolescent/topics/newborn/care_of_preterm.

2. India Newborn action Plan [Internet]. Ministry of health and family welfare, Government of India. Sep 2014 [cited 10 Dec 2016]. Available from: https://www.newbornwhocc.org/INAP_Final.pdf.

3. Kangaroo Mother Care & optimal feeding of low birth weight infants. Operational Guidelines [Internet]. Ministry of health and family welfare. Government of India. Sep 2014. Available from: http://rmncha.in/wp-content/uploads/guidelines_img/1487592695.pdf.

4. Conde-Agudelo A, Diaz-Rossello JL. Kangaroo mother care to reduce morbidity and mortality in low birthweight infants. Cochrane Database Syst Rev. 2016(8):Cd002771.

5. Sloan NL, Ahmed S, Mitra SN, Choudhury N, Chowdhury M, Rob U, et al. Community-based Kangaroo mother care to prevent neonatal and infant mortality: a randomized, controlled cluster trial. Pediatrics. 2008;121(5):e1047-59.

6. Mazumder S, Taneja S, Dalpath SK, Gupta R, Dube B, Sinha B, et al. Impact of community-initiated Kangaroo Mother Care on survival of low birth weight infants: study protocol for a randomized controlled trial. Trials. 2017;18(1):262.

7. Rekha H Udani, Anupa R A Hinduja, Suman Rao P N, Kabra NS. Role of Kangaroo Mother Care in Preventing Neonatal Morbidity in the Hospital and Community: A review article. Journal of Neonatology. 2014;28(4):29-36.

8. Moore ER, Anderson GC, Bergman N, Dowswell T. Early skin-to-skin contact for mothers and their healthy newborn infants. Cochrane Database Syst Rev. 2012(5):Cd003519.

9. Prendergast AJ, Humphrey JH. The stunting syndrome in developing countries. Paediatr Int Child Health. 2014;34(4):250-65.

10. Boundy EO, Dastjerdi R, Spiegelman D, Fawzi WW, Missmer SA, Lieberman E, et al. Kangaroo Mother Care and Neonatal Outcomes: A Meta-analysis. Pediatrics. 2016;137(1).

11. Varendi H, Porter RH, Winberg J. Does the newborn baby find the nipple by smell? Lancet (London, England). 1994;344(8928):989-90.

12. Widstrom AM, Wahlberg V, Matthiesen AS, Eneroth P, Uvnas-Moberg K, Werner S, et al. Short-term effects of early suckling and touch of the nipple on maternal behaviour. Early Hum Dev. 1990;21(3):153-63.

13. Alberts JR. Learning as adaptation of the infant. Acta Paediatr Suppl. 1994;397:77-85.

14. De Carvalho M, Robertson S, Friedman A, Klaus M. Effect of frequent breast-feeding on early milk production and infant weight gain. Pediatrics. 1983;72(3):307-11.

15. Dewey KG, Nommsen-Rivers LA, Heinig MJ, Cohen RJ. Risk factors for suboptimal infant breastfeeding behavior, delayed onset of lactation, and excess neonatal weight loss. Pediatrics. 2003;112(3 Pt 1):607-19.
16. Winberg J. Mother and newborn baby: mutual regulation of physiology and behavior--a selective review. Dev Psychobiol. 2005;47(3):217-29.
17. Uvanas-Moberg K, Arn I, Magnusson D. The psychobiology of emotion: the role of the oxytocinergic system. Int J Behav Med. 2005;12(2):59-65.
18. Dennis CL. Theoretical underpinnings of breastfeeding confidence: a self-efficacy framework. J Hum Lact. 1999;15(3):195-201.
19. Kroenke K, Spitzer RL, Williams JB. The PHQ-9: validity of a brief depression severity measure. J Gen Intern Med. 2001;16(9):606-13.
20. Gjerdingen D, Crow S, McGovern P, Miner M, Center B. Postpartum depression screening at well-child visits: validity of a 2-question screen and the PHQ-9. Annals of family medicine. 2009;7(1):63-70.
21. Norhayati MN, Hazlina NH, Asrenee AR, Emilin WM. Magnitude and risk factors for postpartum symptoms: a literature review. J Affect Disord. 2015;175:34-52.
22. Shidhaye P, Giri P. Maternal depression: a hidden burden in developing countries. Ann Med Health Sci Res. 2014;4(4):463-5.
23. Gelaye B, Rondon MB, Araya R, Williams MA. Epidemiology of maternal depression, risk factors, and child outcomes in low-income and middle-income countries. The lancet Psychiatry. 2016;3(10):973-82.
24. Bigelow A, Power M, MacLellan-Peters J, Alex M, McDonald C. Effect of mother/infant skin-to-skin contact on postpartum depressive symptoms and maternal physiological stress. J Obstet Gynecol Neonatal Nurs. 2012;41(3):369-82.
25. de Alencar AE, Arraes LC, de Albuquerque EC, Alves JG. Effect of Kangaroo mother care on postpartum depression. J Trop Pediatr. 2009;55(1):36-8.
26. Athanasopoulou E, Fox JR. Effects of Kangaroo mother care on maternal mood and interaction patterns between parents and their preterm, low birth weight infants: a systematic review. Infant Ment Health J. 2014;35(3):245-62.
27. Syed S, Ali A, Duggan C. Environmental Enteric Dysfunction in Children. J Pediatr Gastroenterol Nutr. 2016;63(1):6-14.
28. The MAL-ED study: a multinational and multidisciplinary approach to understand the relationship between enteric pathogens, malnutrition, gut physiology, physical growth, cognitive development, and immune responses in infants and children up to 2 years of age in resource-poor environments. Clin Infect Dis. 2014;59 Suppl 4:S193-206.

29. Kosek M, Haque R, Lima A, Babji S, Shrestha S, Qureshi S, et al. Fecal markers of intestinal inflammation and permeability associated with the subsequent acquisition of linear growth deficits in infants. Am J Trop Med Hyg. 2013;88(2):390-6.
30. Pennisi E. Microbiome. The right gut microbes help infants grow. Science. 2016;351(6275):802.
31. Subramanian S, Blanton LV, Frese SA, Charbonneau M, Mills DA, Gordon JI. Cultivating healthy growth and nutrition through the gut microbiota. Cell. 2015;161(1):36-48.
32. Blanton LV, Charbonneau MR, Salih T, Barratt MJ, Venkatesh S, Ilkaveya O, et al. Gut bacteria that prevent growth impairments transmitted by microbiota from malnourished children. Science. 2016;351(6275).
33. Arrieta MC, Stiemsma LT, Amenyogbe N, Brown EM, Finlay B. The intestinal microbiome in early life: health and disease. Front Immunol. 2014;5:427.
34. Thaver D, Zaidi AK. Burden of neonatal infections in developing countries: a review of evidence from community-based studies. Pediatr Infect Dis J. 2009;28(1 Suppl):S3-9.
35. Fransson AL, Karlsson H, Nilsson K. Temperature variation in newborn babies: importance of physical contact with the mother. Arch Dis Child Fetal Neonatal Ed. 2005;90(6):F500-4.
36. Ludington-Hoe SM, Nguyen N, Swinth JY, Satyshur RD. Kangaroo care compared to incubators in maintaining body warmth in preterm infants. Biol Res Nurs. 2000;2(1):60-73.
37. Abouelfettoh A, Ludington-Hoe SM, Burant CJ, Visscher MO. Effect of skin-to-skin contact on preterm infant skin barrier function and hospital-acquired infection. J Clin Med Res. 2011;3(1):36-46.
38. Rath BK, Ghai OP, Bhan MK, Arora NK, Dhar V, Thakkar D, et al. Metoclopramide in lactational failure. Indian Pediatr. 1983;20(5):341-4.
39. WHO. Infant and Young Child Feeding Model Chapter for textbooks for medical students and allied health professionals. Geneva, Switzerland: World Health Organization; 2009.
40. Altuntas N, Turkyilmaz C, Yildiz H, Kulali F, Hirfanoglu I, Onal E, et al. Validity and reliability of the infant breastfeeding assessment tool, the mother baby assessment tool, and the LATCH scoring system. Breastfeed Med. 2014;9(4):191-5.
41. Stable isotope technique to assess intake of human milk in breastfed infants [Internet]. International Atomic Energy Agency. 2010 [cited Oct 2016]. Available from: http://www-pub.iaea.org/books/IAEABooks/8168/Stable-Isotope-Technique-to-Assess-Intake-of-Human-Milk-in-Breastfed-Infants.
42. Ettyang GA, van Marken Lichtenbelt WD, Esamai F, Saris WH, Westerterp KR. Assessment of body composition and breast milk volume in lactating mothers in pastoral communities in Pokot, Kenya, using deuterium oxide. Ann Nutr Metab. 2005;49(2):110-7.
43. Meier PP. Supporting lactation in mothers with very low birth weight infants. Pediatr Ann. 2003;32(5):317-25.

44. Frith AL, Naved RT, Persson LA, Frongillo EA. Early prenatal food supplementation ameliorates the negative association of maternal stress with birth size in a randomised trial. Matern Child Nutr. 2015;11(4):537-49.
45. Benjamin Neelon SE, Stroo M, Mayhew M, Maselko J, Hoyo C. Correlation between maternal and infant cortisol varies by breastfeeding status. Infant Behav Dev. 2015;40:252-8.
46. Takeda S, Kuwabara Y, Mizuno M. Concentrations and origin of oxytocin in breast milk. Endocrinol Jpn. 1986;33(6):821-6.
47. Miller EM, Aiello MO, Fujita M, Hinde K, Milligan L, Quinn EA. Field and laboratory methods in human milk research. Am J Hum Biol. 2013;25(1):1-11.
48. Weaver LT, Arthur HM, Bunn JE, Thomas JE. Human milk IgA concentrations during the first year of lactation. Arch Dis Child. 1998;78(3):235-9.
49. Neville MC, Keller R, Seacat J, Lutes V, Neifert M, Casey C, et al. Studies in human lactation: milk volumes in lactating women during the onset of lactation and full lactation. The American journal of clinical nutrition. 1988;48(6):1375-86.
50. Ballard O, Morrow AL. Human milk composition: nutrients and bioactive factors. Pediatr Clin North Am. 2013;60(1):49-74.
51. Lund CH, Osborne JW, Kuller J, Lane AT, Lott JW, Raines DA. Neonatal skin care: clinical outcomes of the AWHONN/NANN evidence-based clinical practice guideline. Association of Women's Health, Obstetric and Neonatal Nurses and the National Association of Neonatal Nurses. J Obstet Gynecol Neonatal Nurs. 2001;30(1):41-51.
52. Rothman K, Lash T, Greenland S. Modern Epidemiology. Third, Mid-cycle revision edition ed. Philadelphia, PA: Lipincott Williams & Wilkins 2012 December 17, 2012.
53. Andersson T, Alfredsson L, Kallberg H, Zdravkovic S, Ahlbom A. Calculating measures of biological interaction. European journal of epidemiology. 2005;20(7):575-9.
54. Knol MJ, VanderWeele TJ, Groenwold RH, Klungel OH, Rovers MM, Grobbee DE. Estimating measures of interaction on an additive scale for preventive exposures. European journal of epidemiology. 2011;26(6):433-8.

Annexures

Annexure 1. Conceptual framework: Kangaroo mother care – possible biological mechanisms

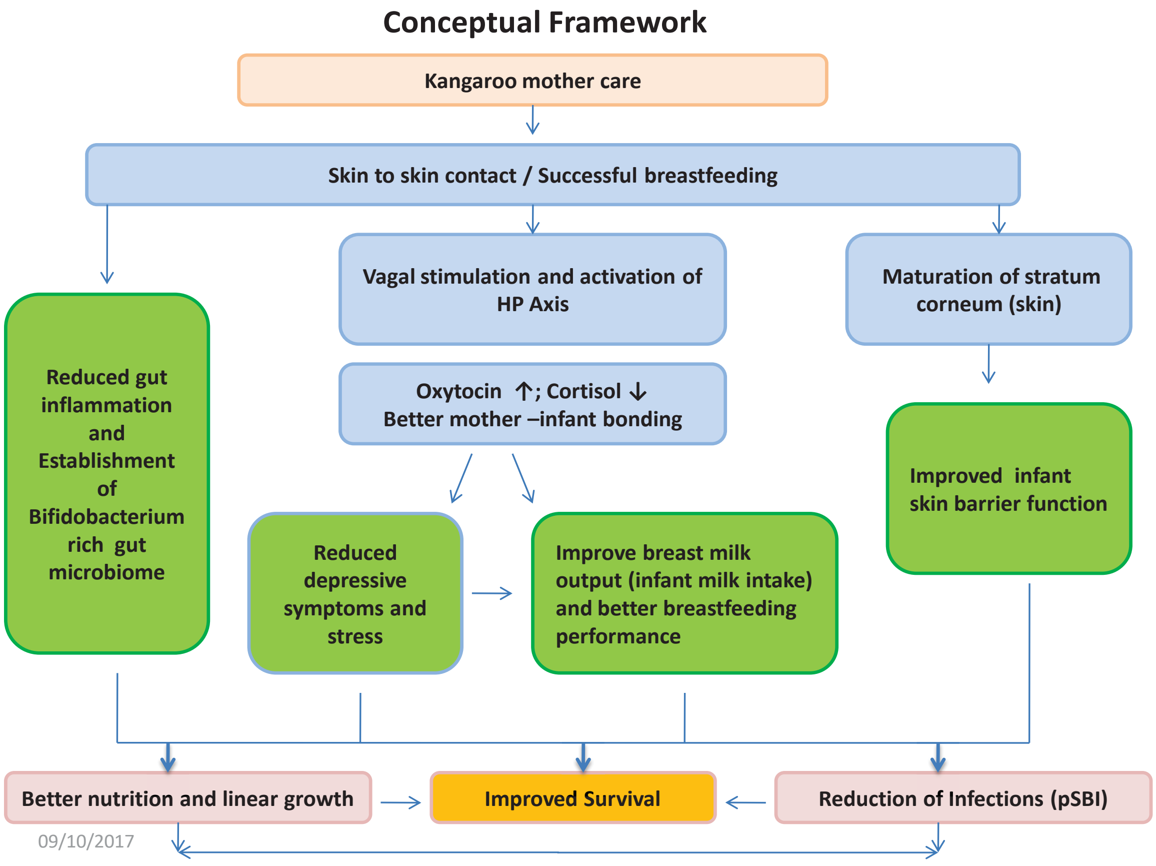

Annexure 2. Implementation strategy for the sub-study

"D0"

LBW mother infant dyad enrolled in the main trial (8-10 enrolments/day)

Randomization by SNOSE, Allocation into intervention and control

Intervention | Control

Inform the sub-study Coordinator

Four subjects selected randomly per day will be enrolled in the sub-study. Only singleton LBW mother-infant dyads will be enrolled.

Intervention | Control

"D4"

Consent for enrolment in the sub-study on D4 by the sub-study Coordinator/ Supervisor

Day of Enrolment in the Sub-study

Visit by sub-study OM team (1 Technical assistant and 1 field worker): 2/3 teams Measure maternal height and weight, ask maternal Dietary history, H/O iron and Ca intake, previous breastfeeding experience, any maternal postpartum illness (e.g. fever)Collect baseline salivary sample from mother and baby and administer 30g of $^{2}H_2O$ drink to mother. Collect stool sample of the baby

"D5"

Collect salivary sample from mother and baby

"D7"

Collect salivary sample from mother and baby

"D11"

Collect salivary sample from mother and baby

"D17"

Collect salivary sample from mother and baby

"D18"

Collect salivary sample from mother and baby
Collect 15 ml breast-milk samples Weigh mother and baby

"D28" of life

Assess IFBAT score, Lactation failure, use of breast-milk substitutes, Administer PHQ-9 questionnaire in mothers

Collect infant and maternal saliva samples and infant stool samples

Annexure 3. Sample Sizes

Infant Breast Milk intake

Sample Size considerations

Standard deviation of breast milk intake in control group at around day 14 (ml/d)	200
Expected increase in Mean (ml/d) in Breast milk output (~10%) [Mean milk output at 0-1 month: 600 ml]	60
Effect size (SD)	0.3
Alpha error (%)	5
Power (1- beta) %	90
Required sample size **per group**	234
**Total sample size required**	468
**TOTAL SAMPLE SIZE APPROVED FOR THIS OUTCOME (including 15% attrition)**	**550**

Ref: Teresa H. M. da Costa et al. How Much Human Milk Do Infants Consume? Data from 12 Countries Using a Standardized Stable Isotope Methodology. J. Nutr. 140: 2227–32, 2010

9/3/2017 8

Maternal Postpartum Depressive symptoms (PHQ 9 ≥10)

Sample Size considerations

Proportion of PP depression in control group	0.19
Expected proportion of PP depression in KMC group (expected 30% relative reduction)	0.133
Estimated risk difference	0.057
Power (1- beta) %	90
Alpha error (%)	5
Required sample size for each arm	874
TOTAL SAMPLE SIZE FOR EACH ARM (including ~10% attrition)	**975**
TOTAL SAMPLE SIZE (including ~10% attrition)	**1950**

Ref: Gelaye B, Rondon MB, Araya R, Williams MA. Epidemiology of maternal depression, risk factors, and child outcomes in low-income and middle-income countries. Lancet Psychiatry 2016;3: 973–82

Stress (Salivary cortisol levels)

Sample Size considerations

Standard deviation of Cortisol in control mothers(ug /dl)	5
Expected Mean difference (ug/dl) in Cortisol levels(~20%) [mean in control mothers7ug/dl]	1.5
Effect size (SD)	0.3
Alpha error (%)	5
Power (1- beta) %	90
Required sample size per group	234
Total sample size required	468
TOTAL SAMPLE SIZE APPROVED FOR THE STUDY (including attrition)	550

Ref: Morelius E, Ortenstrand A, Theodorsson E, Frostell A. A randomised trial of continuous skin-to skin contact after preterm birth and the effects on salivary cortisol, parental stress, depression, and breastfeeding. Early human development. 2015;91(1):63-70.

9/3/2017 24

Gut inflammation

Standard deviation of gut inflammatory markers in controls	1
Expected Mean difference between groups	0.5
Effect size	0.5
Alpha error (%)	5
Power (1- beta) %	90
1 or 2 sided	2
Required sample size per group	84
Total sample size required	168
Total sample size approved for the study including attrition	250
Sample size may be revised after first 100 assays after reviewing the mean and SD of the gut markers	

Stool will be collected from 400 infants at baseline and at 1 month age (ethics approval obtained) from those who provide consent. After the first 100 assays an independent DSMB can look at the data and guide if sample size revision for the gut inflammatory markers are required.

Annexure 4. Table showing schedule of anthropometry / collection of biological specimen[1,2]

**Outcome measured**	**Sample/Procedure**	**Method**	**Total Sample**	Day enrolment	Day 1	Day 3	Day 7	Day 13	Day14	Day 28 (age)
Infant Breast milk intake	Saliva-Mother Infant	FITR[3]/ ELISA	550	√	√	√	√	√	√	-
Depressive symptoms	PHQ-9 (Mother)	Questionnaire	1250	-	-	-	-	-	-	√
Stress (Cortisol)	Saliva-Mother Infant	ELISA	550	-	-	-	-	-	-	√
Gut inflammation	Stool (5g)	ELISA	250	√	-	-	-	-	-	√
Successful BF/Lactation failure	BF assessment	Questionnaire	550	-	-	-	-	-	-	√

[1]The days indicates days after enrolment in the KMC substudy (PhD) unless indicated otherwise

[2]The first four parameters are the primary objectives of the PhD; and the last three parameters are not necessarily a part the PhD

[3]Fourier transform infrared spectophotometry

Annexure 5. Other outputs of this research work

A. Other Objectives of this Research (not necessarily a part of the PhD)

1. To assess the effect of c-KMC on:
    a. Infant gut microbial composition and relative abundance of *Bifidobacterium* at the end of neonatal period
    b. Linear growth velocity and attained length for age Z (LAZ) scores and the prevalence of stunting in the first 6 months of life.
    c. Cumulative incidence of possible serious bacterial infection (PSBI) during the neonatal period and;
    d. Skin maturation assessed through neonatal skin score
2. To study the association between infant milk intake with linear growth and incidence of PSBI
3. To study the association between maternal body composition (% body fat, fat mass and fat free mass) on infant birth weight, breast milk intake and linear growth

B. Tentative titles of other Possible papers that can emerge from this research (not necessarily a part of the PhD- may be included if required)

1. The effect of c-KMC on gut microbial composition in LBW infants in India.
2. Association between maternal postpartum depressive symptoms and stress on infant breast milk intake
3. Association between maternal postpartum depressive symptoms and stress on linear growth
4. Association between gut microbial composition of LBW neonates in which half have received c-KMC and their linear growth in the first six months of life.
5. Effect of c-KMC on neonatal skin condition in LBW infants
6. Association of maternal body composition with infant birth weight, breast milk intake and linear growth (from secondary data that will be generated from this work).

Responsibilities of Supervisors

Halvor Sommerfelt is the principal supervisor from University of Bergen, responsible for guiding the PhD candidate throughout the program.

Maharaj Kishan Bhan (Adjunct Professor 20% position at the University of Bergen, and National Science Professor in India) will be the Internal Co-Supervisor and will be responsible for guiding the PhD student, providing necessary feedback on protocol/ research plan/ manuscripts and will help the candidate to complete the research and successfully submit the PhD thesis.

Nita Bhandari and Rajiv Bahl will provide necessary support to the PhD candidate during the PhD programme and will also provide necessary feedback on protocol/ research plan/ manuscripts to ensure completion of the PhD research successfully by the candidate within time.

Plan of Analysis
BS-PhD manuscript

Effect of community-initiated KMC on maternal postpartum depressive symptoms and stress - a randomized controlled trial

Introduction

This plan of analysis is described for a randomised controlled trial conducted to evaluate the impact of community-initiated Kangaroo Mother Care (ciKMC) in low birth weight mother-infant dyads on maternal postpartum depressive symptoms and maternal salivary cortisol levels (a marker of stress). This document describes the methods of data analysis.

Hypothesis tested

Community-initiated Kangaroo mother care leads to reduction in the prevalence of postpartum moderate to severe depressive symptoms (prevalence ratio ≤0.7) and reduction in salivary cortisol levels (at least 0.3 SD in mean values) as a marker of maternal stress.

Objectives

To estimate the effect of community initiated Kangaroo mother care during the neonatal period in LBW infants on

- prevalence of moderate to severe postpartum depressive symptoms (Patient Health Questionnaire-9 score ≥10) and
- stress (measured by salivary cortisol levels) at the end of the neonatal period

Outcomes

- Primary: Prevalence of moderate to severe maternal depressive symptoms (PHQ-9 score ≥10)
- Secondary:
    - Mean salivary cortisol levels (in ug/dl)
    - Prevalence of Mild depressive symptoms (PHQ-9 score 5-9)
    - Proportion of mothers reporting suicidal ideation
    - Mean Patient Health Questionnaire-9 (PHQ-9) scores

Definition of postpartum depression

As per DSM-IV/V (Am Asso. of Psy.) an episode of depression is specified as having a postpartum onset if it occurs *within the first 4 weeks* after delivery (American Psychiatric Association) with the argument that onset of such an episode with onset later than 4 weeks could not easily be identified as being related to childbirth.

Validity of the PHQ-9 tool used in the study

- PHQ-9 is a screening tool that has been previously validated against DSM IV criteria (gold standard) to diagnose Post-partum depression (PPD) and is found to be highly specific.

Plan of Analysis
BS-PhD manuscript

Effect of community-initiated KMC on maternal postpartum depressive symptoms and stress - a randomized controlled trial

- Gjerdingen D et al in 2009 in a study in 506 mothers demonstrated that the *sensitivity and specificity* of a PHQ-9 score ≥ 10 to diagnose major depression as compared to DSM IV criteria is 75% and 91%, respectively, for diagnosing PPD within 1 month of child birth. In another study by Arroll et al (2010) in 2642 participants where the sensitivity and specificity of a PHQ-9 score ≥ 10 to diagnose major depression as compared to DSM IV criteria was 74% and 91% respectively.
- Given that the tool used is a screening tool we have opted to use the term "depressive symptoms". The categorizations of minimal, mild, moderate to severe are according to previous literature (Kronke et al, 2001). Minimal, Mild and Moderate to severe depressive symptoms are defined by PHQ-9 scores 0 to 4, 5 to 9 and ≥10, respectively

Process for PHQ-9 assessments

- **Tool:** Validated PHQ 9 questionnaire (hindi version). This was the same hindi form that was pre-tested and used in one of our previous studies.
- **Process:** Assessment was done at home by an independent team at the *end of neonatal period* i.e. day 28 (window period was +7 days) (Though the workers are part of the intervention delivery team, it was ensured that the workers assessed PHQ9 in mothers belonging to a different area (PHC) where they were not involved in intervention delivery so that the observations are independent.) If door was locked or the mother was unavailable and her date of availability was uncertain, she / family was contacted either by home visit or phone for at least 3 times within the window period.
- **Training:** Workers were trained to conduct interview for PHQ 9 and fill the PHQ 9 questionnaire following the KMC-GCC form filling guidelines. The workers were reassessed and retrained by a faculty of the department of clinical psychology of AIIMS, New Delhi. The PI, Coordinators were also trained for assessing PHQ 9. Training logs are maintained.
- **Quality checks:** The PI of the study conducted random visits in 1-2% of the PHQ 9 assessments and checked whether the PHQ 9 scores matched when scored independently. Also around 2% of the interviews are being recorded after taking consent for recording.
- **Advise for consultation:** All Mothers, irrespective of any group, who are having scores >=10 were advised to consult a nearby physician at the earliest for further advise. A standard message is given to the family. Facilitation was done wherever possible.

Sample Size:

Plan of Analysis
BS-PhD manuscript

Effect of community-initiated KMC on maternal postpartum depressive symptoms and stress - a randomized controlled trial

1950 mothers (975 in each group) was enrolled which was calculated to be sufficient to detect a 30% relative reduction in the prevalence of moderate to severe postpartum depressive symptoms (PHQ 9 ≥10), assuming a baseline prevalence of 19%, and considering attrition of 10%

A subsample i.e. 550 mothers were enrolled for saliva collection to measure salivary cortisol levels (biomarker of stress). This was calculated to be sufficient to detect to detect a 0.3 SD i.e. 1.5 ug/dl decrease in the mean saliva cortisol levels considering attrition of 15%. 2 Saliva samples were collected in the morning before 12.00 hours before and after breastfeeding.

Plan of Analysis

The analysis will be done using STATA version 14 (Stata Corporation, College Station, TX). All analysis will be performed on intent to treat basis.

Group wise comparison will be done for distribution of baseline features such as

- household characteristics (wealth quintile, religion, caste, type of family, number of family members)
- maternal and paternal characteristics (mother's age, mother's education, maternal occupation, father's age, father's education and occupation)
- birth related characteristics (place and type of delivery, birth order, parity) and
- infant characteristics (sex, birth weight, gestational age)

Proportion who initiated breastfeeding within 1 hour, exclusive breastfeeding, hospitalizations (at the 1 month visit) will also be presented by group.
Gestational age will be calculated from ultrasonography (USG) report, hospital records or maternal recall, whichever available, in the given order of preference. Proportion of women with available antenatal USG will be reported.

The distribution of the continuous outcomes such as (maternal salivary cortisol concentration, PHQ-9 scores) will be examined by using histogram and normal probability plot. Skewness and kurtosis coefficient will be measured. If skewness coefficient tends to zero and kurtosis coefficient tends to 3, distribution will be normal. For normal distribution, mean and standard deviation (SD) will be reported for quantitative variables. Median and interquartile range (IQR) will be reported for non-normal distribution. For non-normal continuous outcomes appropriate transformation (log or others) will be done before applying the tests of association or we will use non-parametric tests such as Wilcoxon rank-sum test will be performed for comparison between study arms.

The proportion of minimal, mild and moderate to severe depressive symptoms as defined by the PHQ-9 scores will be presented by group.

Plan of Analysis
BS-PhD manuscript

Effect of community-initiated KMC on maternal postpartum depressive symptoms and stress - a randomized controlled trial

To examine effect of ciKMC on maternal moderate to severe depressive symptoms multivariable logistic regression analysis or analyses with generalized linear models of the binomial family with a log link shall be done. Additionally, multinomial logistic regression analysis will be done to examine effect of ciKMC on prevalence of categories of maternal depressive symptoms viz. minimal (reference group), mild, and moderate to severe depressive symptoms.

For estimating effect of ciKMC on continuous outcomes i.e. maternal salivary cortisol concentration and mean PHQ-9 scores multivariable linear regression will be done if distribution is normal. If distributions are skewed, appropriate transformation (log or others) will be done before conducting linear regression. Alternatively, we will use non-parametric tests such as Wilcoxon rank-sum test for comparison of these outcomes between study arms.

For all the analyses, univariable regression analysis will be done as an initial step to assess the effect of ciKMC on the outcomes considered. This will be followed by multivariable regression analysis after including potential confounding variables in the model, defined as variables which are unequally distributed between the two groups (relative difference of >10%).

Potential interaction between KMC and other variables will be examined by including interaction terms in the multivariable regression models. Stratified analysis will be presented and any biologic interaction will be estimated with the relative excess risk due to interaction (RERI).

Planned Sub-group analysis:

1) By preterm vs. term birth
2) Birth weight +/- 2 kg if it does not substantially correlate with term vs. preterm birth.
