# Supplementary material for: Effect of Community-Initiated Kangaroo Mother Care on Postpartum Depressive Symptoms and Stress Among Mothers of Low-Birth-Weight Infants: A Randomized Clinical Trial
Source: JAMA Netw Open. 2021 Apr 22;4(4):e216040. doi: 10.1001/jamanetworkopen.2021.6040 (PMC8063066; doi:10.1001/jamanetworkopen.2021.6040)
Supplement: Supplement 2. — eTable 1. Practice of Kangaroo Mother Care Intervention Components and Home Visits Among Study Participants eTable 2. Effect of ciKMC on Different Categories of Postpartum Depressive Symptoms Among Mothers at the End of the Neonatal Period eTable 3. Proportion of Mothers With Postpartum Depressive Symptoms in Control and ciKMC Arm Using Different PHQ-9 Cutoff Scores [file jamanetwopen-e216040-s002.pdf]

Supplemental Online Content

Sinha B, Sommerfelt H, Ashorn P, et al. Effect of community-initiated kangaroo mother care on postpartum depressive symptoms and stress among mothers of low-birth-weight infants: a randomized clinical trial. *JAMA Netw Open*. 2021;4(4):e216040. doi:10.1001/jamanetworkopen.2021.6040

eTable 1. Practice of Kangaroo Mother Care Intervention Components and Home Visits Among Study Participants

eTable 2. Effect of ciKMC on Different Categories of Postpartum Depressive Symptoms Among Mothers at the End of the Neonatal Period

eTable 3. Proportion of Mothers With Postpartum Depressive Symptoms in Control and ciKMC Arm Using Different PHQ-9 Cutoff Scores

This supplemental material has been provided by the authors to give readers additional information about their work.

© 2021 Sinha B et al. *JAMA Network Open.*

eTable 1. Practice of Kangaroo Mother Care Intervention Components and Home Visits Among Study Participants

Variables	Control arm (N=852)	ciKMC arm (N=974)
Skin to Skin contact (SSC)		
Any SSC received from enrolment to day 28 of birth	33 (3.8)	971 (99.7)
Days with SSC		
Mean (SD)	1.1 (5.2)	27.5 (2.8)
Median (IQR)	0 (0-0)	28 (28-28)
Duration of SSC per day in hour		
Mean (SD)	0.2 (1.4)	12.0 (3.7)
Median (IQR)	0 (0-0)	13 (10-15)
Breastfeeding		
Time of breastfeeding initiation in hour		
Mean (SD)	4.4 (10.0)	4.0 (8.6)
Median (IQR)	1 (0, 3)	1 (0, 3)
Breastfeeding initiated within ≤1 hour of birth	462 (54.2)	516 (53.0)
Exclusive Breastfeeding at 28 days of birth	486 (57.0)	859 (88.2)
Home visits by ASHA[1] (as reported by mother)		
At least once during neonatal period	705 (82.7)	796 (81.7)
Median number of ASHA visits per child during the neonatal period (IQR)	2 (1-3)	2 (1-3)

[1]Accredited Social Health Activist (ASHA) is a community health worker instituted by the government of India's Ministry of Health and Family Welfare.

© 2021 Sinha B et al. *JAMA Network Open.*

eTable 2. Effect of ciKMC on Different Categories of Postpartum Depressive Symptoms Among Mothers at the End of the Neonatal Period[1,2]

Outcome Variable	Control arm<br><br>N=852	ciKMC arm<br><br>N=974	Unadjusted	Adjusted[3]	*P* value
	**n (%)**	**n (%)**	**RR (95% CI)**	**RR (95% CI)**	
None or Minimal depressive symptoms	577 (67.7)	680 (69.8)	Baseline	Baseline	
Mild depressive symptoms	159 (18.7)	189 (19.4)	1.01 (0.79 to 1.28)	1.00 (0.79 to 1.26)	0.91
Moderate-to-severe depressive symptoms	116 (13.6)	105 (10.8)	0.77 (0.58 to 1.02)	0.73 (0.54 to 0.98)	0.03

[1]Results from multinomial regression
[2]ciKMC is community-initiated Kangaroo mother care
[3]Adjusted for birth order categories and accounted for household clustering.

© 2021 Sinha B et al. *JAMA Network Open.*

eTable 3. Proportion of Mothers With Postpartum Depressive Symptoms in Control and ciKMC Arm Using Different PHQ-9 Cutoff Scores[1]

PHQ-9 cut off score	Control arm<br><br>N (%)	ciKMC arm<br><br>N (%)	Unadjusted Relative Risk[2] (95%CI)	Adjusted Relative Risk[3] (95% CI)
≥2	482 (56.6)	540 (55.4)	0.98 (0.90 to 1.06)	0.97 (0.90 to 1.05)
≥4	340 (39.9)	366 (37.6)	0.94 (0.84 to 1.06)	0.93 (0.83 to 1.04)
≥6	229 (26.9)	242 (24.8)	0.92 (0.80 to 1.08)	0.91 (0.78 to 1.06)
≥8	171 (20.1)	165 (16.9)	0.84 (0.69 to 1.02)	0.82 (0.68 to 0.99)
≥10	116 (13.6)	105 (10.8)	0.79 (0.62 to 1.01)	0.75 (0.59 to 0.96)
≥12	84 (9.9)	76 (7.8)	0.79 (0.59 to 1.06)	0.75 (0.56 to 1.02)
≥14	53 (6.2)	41 (4.2)	0.68 (0.45 to 1.00)	0.63 (0.42 to 0.94)
≥16	33 (3.9)	30 (3.1)	0.80 (0.49 to 1.29)	0.73 (0.45 to 1.20)
≥18	21 (2.5)	15 (1.5)	0.62 (0.32 to 1.20)	0.58 (0.30 to 1.14)
≥20	14 (1.6)	7 (0.7)	0.43 (0.18 to 1.08)	0.40 (0.16 to 1.00)
≥22	3 (0.4)	0 (0.0)	-	-
≥24	3 (0.4)	0 (0.0)	-	-
≥26	1 (0.1)	0 (0.0)	-	-

[1]ciKMC is community-initiated Kangaroo mother care
[2] Unadjusted risk of having higher PHQ-9 scores (based on the given cut-off) in intervention arm mothers compared to control arm mothers.
[3]Adjusted for birth order categories and accounted for household clustering.

© 2021 Sinha B et al. *JAMA Network Open.*
