# Supplementary material for: Effect of Community-Initiated Kangaroo Mother Care on Postpartum Depressive Symptoms and Stress Among Mothers of Low-Birth-Weight Infants: A Randomized Clinical Trial
Source: JAMA Netw Open. 2021 Apr 22;4(4):e216040. doi: 10.1001/jamanetworkopen.2021.6040 (PMC8063066; doi:10.1001/jamanetworkopen.2021.6040)
Supplement: Supplement 3. — Data Sharing Statement [file jamanetwopen-e216040-s003.pdf]

Data Sharing Statement

Sinha. Effect of Community-Initiated Kangaroo Mother Care on Postpartum Depressive Symptoms and Stress Among Mothers of Low-Birth-Weight Infants. *JAMA Netw Open*. Published April 22, 2021.
doi:10.1001/jamanetworkopen.2021.6040

Data
Data available: No

Additional Information
Explanation for why data not available: The primary custodian of the data is Centre for Health Research and Development, Society for Applied Studies (CHRD SAS), India. As per the Institutional policy de-identified data will be made available on request for the purpose of checking consistency or supporting the analyses presented in this scientific manuscript.
